# Experimental and *In Silico* Analysis of TEM β-Lactamase Adaptive Evolution

**DOI:** 10.1021/acsinfecdis.2c00216

**Published:** 2022-11-15

**Authors:** Melissa Standley, Vincent Blay, Violeta Beleva Guthrie, Jay Kim, Audrey Lyman, Andrés Moya, Rachel Karchin, Manel Camps

**Affiliations:** # Department of Microbiology and Environmental Toxicology, University of California, Santa Cruz, California95064, United States; ‡ Institute for Integrative Systems Biology (I2Sysbio), Universitat de València and Spanish Research Council (CSIC), 46980Valencia, Spain; § Department of Biomedical Engineering and Institute for Computational Medicine, The Johns Hopkins University, Baltimore, Maryland21218, United States; ∥ Foundation for the Promotion of Sanitary and Biomedical Research of Valencia Region (FISABIO), 46021Valencia, Spain; ⊥ CIBER in Epidemiology and Public Health (CIBEResp), 28029Madrid, Spain

**Keywords:** evolution, fitness landscape, epistasis, contingency, selection, antibiotic resistance

## Abstract

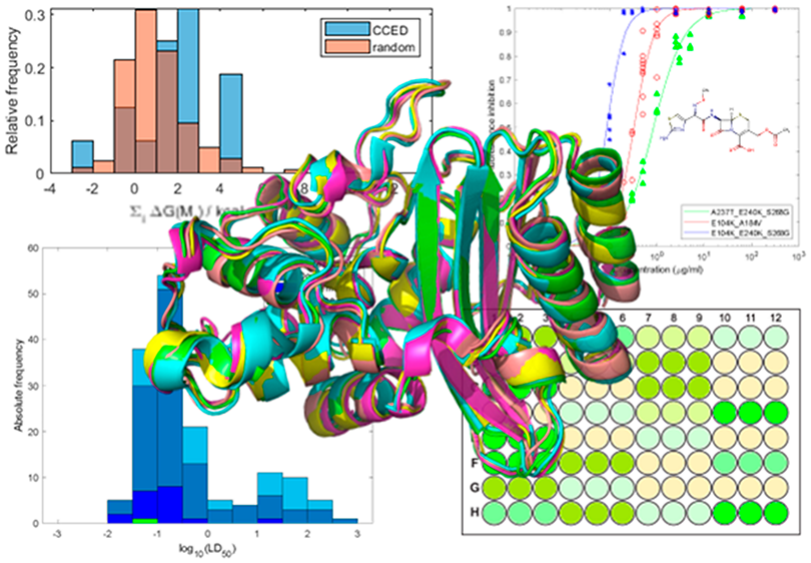

Multiple mutations often have non-additive (epistatic) phenotypic effects. Epistasis is of fundamental biological relevance but is not well understood mechanistically. Adaptive evolution, i.e., the evolution of new biochemical activities, is rich in epistatic interactions. To better understand the principles underlying epistasis during genetic adaptation, we studied the evolution of TEM-1 β-lactamase variants exhibiting cefotaxime resistance. We report the collection of a library of 487 observed evolutionary trajectories for TEM-1 and determine the epistasis status based on cefotaxime resistance phenotype for 206 combinations of 2–3 TEM-1 mutations involving 17 positions under adaptive selective pressure. Gain-of-function (GOF) mutations are gatekeepers for adaptation. To see if GOF phenotypes can be inferred based solely on sequence data, we calculated the enrichment of GOF mutations in the different categories of epistatic pairs. Our results suggest that this is possible because GOF mutations are particularly enriched in sign and reciprocal sign epistasis, which leave a major imprint on the sequence space accessible to evolution. We also used FoldX to explore the relationship between thermodynamic stability and epistasis. We found that mutations in observed evolutionary trajectories tend to destabilize the folded structure of the protein, albeit their cumulative effects are consistently below the protein’s free energy of folding. The destabilizing effect is stronger for epistatic pairs, suggesting that modest or local alterations in folding stability can modulate catalysis. Finally, we report a significant relationship between epistasis and the degree to which two protein positions are structurally and dynamically coupled, even in the absence of ligand.

A basic tenet of modern biology is that genotypes determine phenotypes, which in turn affect fitness. The non-additive phenotypic impact of mutations is a phenomenon known as epistasis.^1^ Epistasis comes in different flavors depending on whether they result in a further increase in fitness relative to the additive case (positive epistasis) or in a decrease (negative epistasis). Sometimes the direction of a given mutation on fitness can be reversed in the presence of a second mutation (sign epistasis). On rare occasions, two mutations that have a negative impact on fitness on their own can improve fitness when they co-occur (reciprocal sign epistasis).

Epistatic interactions are key factors shaping evolution because they directly impact the probability of mutational pathways. Sign and reciprocal sign epistatic interactions are the ones with the largest impact on local fitness, as they can render whole mutational pathways inaccessible.^2−4^ Understanding epistasis is therefore relevant for modeling evolutionary processes, such as random genetic drift, recombination, divergence,^5^ and genetic robustness,^6^ and for understanding how proteins evolve new biological activities.^7,8^

Our understanding of how epistatic interactions contribute to phenotypic outcomes is very limited, especially when it comes to higher-order interactions.^9,10^ The main challenges for the study of epistatic interactions are the astronomical number of possible combinations in a given protein and the difficulty of profiling epistasis at scale. Therefore, the data available is partial, either restricted to a few positions or restricted to specific types of combinations.^4^ Despite these limitations, some organizing principles are emerging, such as (a) the fitness landscape outline most frequently has intermediate ruggedness, (b) mutations with a significant effect tend to show greater ruggedness (*size effect*), (c) incorporating additional mutations produces diminishing returns, and (d) low- and high-fitness effect mutations are qualitatively distinct.^4,11,12^ Moreover, protein conformation is known to be a key contributor because it depends on its folding energy, which is impacted by mutations.^13,14^

β-Lactamases are a family of enzymes that hydrolyze lactam compounds, conferring resistance to a range of β-lactam antibiotics in clinical use. The wild-type (WT) of one of these β-lactamases, TEM-1, has poor activity against synthetic cephalosporins because bulky side groups cause steric hindrance in its active site.^15^ Under selective pressure, TEM-1 evolves mutants with the ability to hydrolyze synthetic cephalosporins.^16^ These mutants (which typically involve between 1 and 4 mutations) are known as extended-spectrum β-lactamase (ESBL) TEM mutants.

The evolution of ESBL mutants of TEM β-lactamase in the laboratory represents a tractable experimental system for the study of adaptation, that is, the evolution of new biochemical activities, and of epistasis. Cefotaxime is one of the cephalosporins of choice in these directed evolution experiments. Using cefotaxime resistance as a proxy for genetic adaptation greatly facilitates the high-throughput phenotypic characterization of mutants.^17,18^ The fitness landscape of TEM-1 β-lactamase evolution under cefotaxime selection is rugged, with several accessible pathways toward high fitness.^8,19,20^

To better understand the principles underlying epistasis in the context of genetic adaptation, we produced a large-scale empirical cefotaxime resistance landscape of TEM-1 mutants involving combinations of 2 or 3 mutations under adaptive selective pressure. We detected epistatic interactions for 95 of the 206 characterized combinations and found that epistatic pairs were highly enriched for gain-of-function (GOF) mutations, particularly negative and reciprocal sign pairs. Using the software FoldX to estimate the effects of mutations on TEM-1 folding stability, we found that epistatic pairs tend to be thermodynamically unstable but that the instability of adaptive mutation trajectories is limited by the free energy of folding of the protein. Finally, we present early attempts at modeling epistasis based on the degree to which two protein positions are structurally and dynamically coupled.

## Results and Discussion

Epistasis is a process of fundamental biological relevance whose mechanistic basis is largely unknown. In order to discern patterns that provide mechanistic insights into this phenomenon, experimental data is limiting. In this work, we generate a landscape of TEM-1 cefotaxime resistance. We selected this well-established model of adaptive evolution because the evolution of new biochemical activities is known to be enriched for epistatic interactions.^21^ To inform and complement these experiments, we used reported data from natural or experimental evolution, following a long tradition of merging directed evolution experiments with clinical data to make inferences about adaptive evolutionary landscapes.^18,22,23^

### Compilation of a Database of Observed Evolutionary Trajectories

We collected 487 TEM mutants reported from the clinic and from directed evolution experiments. In addition to the mutants already reported in ref (18), we added 37 cefotaxime-selected mutants listed in ref (17) and 36 additional ones corresponding to additional cefotaxime directed evolution experiments starting with WT, I173V, M182T, A224V, M182 A224V, E104K M183T, and G238S TEM sequences (M. Salverda, personal communication). We called this database CCED, for Combined Clinical and Experimental Database.

Of the 487 sequences included in this database, 284 displayed the extended spectrum phenotype (2be), 52 were resistant to β-lactamase inhibitors, and 11 displayed both inhibitor resistance and the extended spectrum phenotype (2ber). Additionally, 60 β-lactamases had unassigned phenotypes. 117 broad spectrum mutants were also included because they are enriched for compensatory mutations that overlap with those seen during adaptation and in some cases set the stage for adaptation.^24,25^ The mutations, with the corresponding sources, are listed in the supporting files SI01.xlsx (clinical) and SI02.xlsx (experimental). The mutation frequency, by position, in our database is shown in Figure S1.

For the rest of this work, CCED is assumed to represent the evolution of TEM under positive (largely ESBL and β-lactamase inhibitor-resistance) selection. This library was used to inform our approach for exploring the empirical landscape of cefotaxime resistance, to place the results in the context of observed evolutionary trajectories, and to model the thermodynamic constraints of genetic adaptation.

### Partial Cefotaxime Resistance Landscape of TEM-1 β-Lactamase

To be able to model epistasis in the context of genetic adaptation, we set out to produce a large-scale empirical cefotaxime resistance landscape of TEM-1 mutants. The comprehensive generation of an empirical fitness landscape is not feasible because of the astronomically large number of possible combinations. To guide our exploration of sequence space, we decided to focus on 17 positions which appear to be under adaptive selection based on their representation in CCED (Figure S1) and that are consistent with omega value calculations by our group.^18^ These mutations include a wide range of frequencies in CCED to maximize their representativity (Figure S1). While our guided approach admittedly biases our exploration of the resistance landscape, it also makes it more directly relevant to observed mutational trajectories and further enriches our analysis for the presence of epistatic interactions. This enrichment is necessary to ensure a good representation of different categories of epistatic interactions, which is essential to enable us to model epistasis.

We established the presence and type of epistasis by comparing the IC_50_ values of our test pairs to the expected IC_50_ assuming a linearly additive effect of their constitutive mutations. More specifically, for every pair of mutations (M1 and M2), we defined a *z*-test that compares the experimental IC_50_ of the double mutant (M1 + M2) to the one that would be expected if mutations M1 and M2 had linearly additive effects (see eq 4, below). Note that, in eq 4, M1 is a single mutant but M2 can be a single or a complex mutant. Interactions found to deviate significantly from the expected additive effect were considered epistatic, and a specific type of epistasis was assigned (see Methods).

The dose–response curves for each of the single mutants at the 17 positions included in our study are shown in Table 1. For each position, we tested the amino acid substitutions most frequent in the CCED database (and sometimes other substitutions also found in CCED, marked with an asterisk in Table 1). To generate these dose–response curves, we used a plasmid (pGFPck) bearing both the β-lactamase *Bla*_*TEM-1*_ gene and a Cycle 3 GFP gene^26^ that we used to track growth by measuring fluorescence. Using fluorescence rather than OD substantially increased the dynamic range of the assay and allowed us to normalize our data to plasmid copy number (one of the main determinants of protein expression in recombinant vectors), as different mutants consistently produced different fluorescence levels in the “no-drug” control, indicating vector-dependent variation.

**Table 1:** Cefotaxime IC_50_ of Different Single Mutants and of the Corresponding Wild-Type TEM-1^a^

variant	IC_50_(μg mL^–1^)	*z*-statistic	*p*-value
G238S	21	43.5	0.00
R164H	0.42	12.3	0.00
E104K	0.22	8.4	0.00
M182T	0.14	4.3	9.21 × 10^–6^
E240K	0.13	3.7	9.46 × 10^–5^
D254G	0.15	3.6	1.38 × 10^–4^
A237T	0.12	3.6	1.77 × 10^–4^
I173V	0.12	2.8	2.89 × 10^–3^
H153R	0.12	2.6	4.09 × 10^–3^
A184V	0.10	2.2	1.50 × 10^–2^
T265M	0.10	1.7	4.65 × 10^–2^
*A237G	0.10	1.3	1.00 × 10^–1^
S268G	0.087	1.0	1.60 × 10^–1^
N175I	0.077	0.1	4.72 × 10^–1^
WT	0.076	0.0	5.00 × 10^–1^
L21F	0.067	–0.8	7.85 × 10^–1^
A224V	0.063	–0.9	8.05 × 10^–1^
*R164S	0.000019	–1.5	9.36 × 10^–1^
Q39K	0.021	–4.7	1.00
R275L	0.026	–5.1	1.00

a Most frequent mutations at each position are used by default; additional alternative mutations are indicated with *.

All of the 17 single TEM mutants were generated, transformed, and grown in 96-well plates in the presence of increasing concentrations of cefotaxime (see Methods). We extracted IC_50_ values from the fluorescence data (Table S1) and used a two-parameter sigmoidal model fitted to each dose–response curve to infer the IC_50_ for cefotaxime, that is, the cefotaxime concentration causing a 50% reduction in fluorescence in a specific mutant (see example in Figure S2). Table 1 lists the IC_50_ for cefotaxime (in μg mL^–1^), the *z*-statistic (the statistical difference in log(IC_50_) to TEM-1, which was determined using the *z*-test described in eq 2, below), and the *p*-values. Figure S2b shows a summary of all the IC_50_ values fitted.

The single point mutations analyzed tended to increase cefotaxime resistance. Although most mutations had a modest effect, three mutants stood out for a dramatic increase in IC_50_ for cefotaxime: G238S, R164H, and E104K. G238S and R164H are the earliest mutations to be fixed both in the laboratory^17^ and in nature.^27^ Their large phenotypic impact likely gives them a major competitive advantage when multiple mutants are present in the population.^28,29^ These two mutations are largely mutually exclusive due both to contingency (mutations in positions 164 and 238 are phenotypically redundant) and to reciprocal sign epistasis (the double mutant has a lower level of resistance than either single mutant).^13^

We also see that, when two different amino acid substitutions for a given position are tested (A237S vs A237T, R164H vs R164S), only the most frequent one in CCED has a substantial effect on cefotaxime resistance on its own in our analysis. This implies that less frequent substitutions may be more dependent on a network of interactions to produce a large increase in resistance, consistent with their lower frequency^18,27^ or the fact that their selection may have been driven by a different lactam antibiotic (R164S primarily confers resistance to ceftazidime^30^) or by a lower antibiotic concentration.^31^

Next, we determined the IC_50_ for a total of 206 TEM mutants—101 simple ones (i.e., involving 2 mutations) and 105 complex ones (i.e., involving 3 mutations). The results of the test are shown in Table S2. Using a conservative significance level to account for multiple testing (*p* < 0.0005), our method detected 95 epistatic interactions. Of these 95 detected epistatic interactions, 29 involve simple pairwise interactions, including 18 previously reported ones; of these 18 epistatic pairs, only 2 are inconsistent with previous reports (Table S3). Figure S3 illustrates the location of pairs of positive epistatic mutations and negative epistatic mutations on a structural model of TEM-1. Both types of interactions are found across a broad range of distances, highlighting the complexity of this phenomenon.

### Contribution of Epistatic Interactions to TEM Evolution Driven by - Selection

To investigate the contribution of the epistatic interactions identified in our partial empirical resistance map to the shaping of TEM evolution under selective pressure, we looked at their representation in our CCED mutant database. For every β-lactamase variant in our CCED mutant database, we broke down all possible pairs of mutations and matched them to the 29 simple pairs identified as epistatic in Table S2. The results are included in the file SI04.xlsx. Notice that, for a given mutant, we may identify more than two interactions as epistatic. For instance, in the mutant G238S_E240K_T265M_R275L (entry TEM-68 in file SI04.xlsx), two pairs (G238S_E240K and G238S_T265M) would show positive epistasis, whereas a third pair (G238S_R275L) would show sign epistasis.

**Figure 1:**
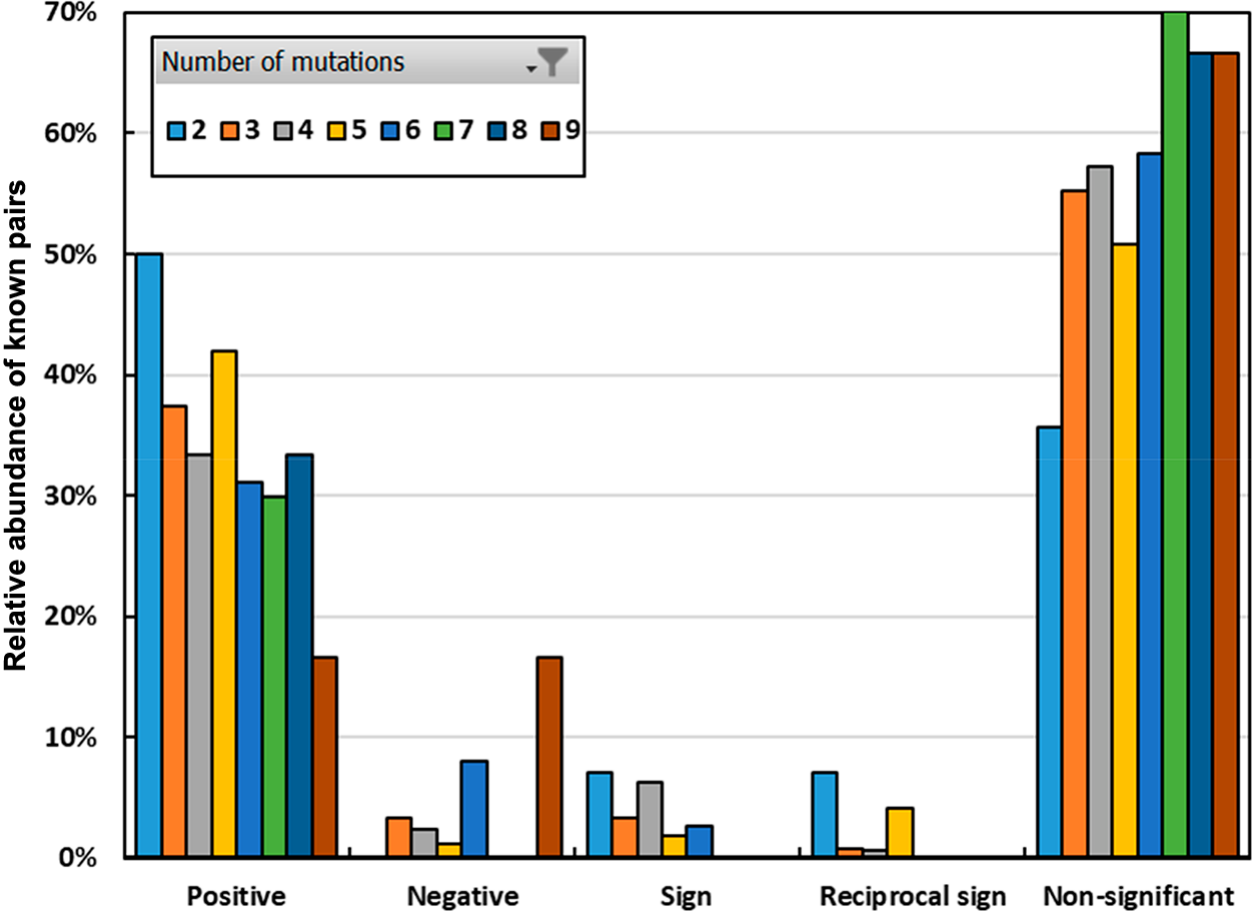
Representation of interactions between mutations in the CCED database of clinical and experimental isolates, broken down by type of interaction and number of mutations in the mutant where the pair was observed. The types of interactions are determined experimentally in the first part of this work (Table S2).

A summary of the results is displayed in Figure 1, showing the representation of each epistatic category relative to the complexity of the TEM mutant (from *n* = 2 to *n* = 9 mutations). We note that, with increasing complexity of the mutants, the proportion of epistatic pairs decreases and that of non-significant pairs increases. This trend is expected because the number of pairs that are the result of our combinatorial analysis but not directly selected increases with the complexity of the mutant, and most random pairs are expected to be non-epistatic. Thus, as the number of possible combinations increases, the likelihood that one pair of mutations therein is epistatic decreases.

The one exception to this trend are negative interactions, which are more frequently observed with increased mutant complexity. This observation is consistent with reports highlighting the importance of genetic drift as a facilitator of adaptation in the long term on rugged fitness landscapes.^32,33^ Possible mechanisms to explain the increased prevalence with increased mutant complexity include two originally negative mutations that may have changed in sign in the presence of a new mutation, or that have been compensated in the new background, or a combination of both.^10^

### Comparison to Large-Scale Empirical Epistasis Studies Reported for TEM-1 Wild-Type Activity

A number of recent large-scale fitness landscape studies reported TEM-1 epistasis in the context of ampicillin selection.^34,35^ While highly valuable, these studies represent a fundamentally different model system because it is hard to improve catalysis on an optimal substrate, so it essentially measures losses in function and compensatory mutations. Loss of function is much more frequent than gain of function and, therefore, tends to be less specific. To illustrate this effect, we performed a direct comparison of epistasis under ampicillin selection and under cefotaxime selection for a specific mutant, TEM-15 (E104K_G238S), using the data reported in ref (36). Averaging the epistasis values for each position (since multiple mutants in each position were characterized), we calculated the correlation between the ampicillin and cefotaxime datasets to be *R*^2^ = 0.69. However, when we compare the average epistasis value for each position of the β-lactamase amino acid sequence (Figure S4), we find that the correlation between ampicillin and cefotaxime epistasis is driven by mutations that non-specifically decrease enzymatic activity. Both ampicillin and cefotaxime identify the critical residues for catalysis: positions 44 to 47, positions 64 to 76, positions 130 to 139, positions 228 to 237, and positions 244 to 252, implying that values outside these positions are different depending on the selection. Indeed, the residues that show the strongest epistasis under cefotaxime selection include three positions known to be critical for adaptation (positions 104, 238, and 240) as well as immediate neighbors (241 and 198) (Figure S4).

### Representation of GOF Mutations in Epistatic Interactions

Mutations that confer a new biochemical activity by themselves (GOF mutations) enable adaptation and define adaptive trajectories.^17,37,38^ Their identification is therefore crucial to build predictive models of drug resistance based on sequencing data. Establishing GOF status requires measuring fitness. The IC_50_ values that we determined are a measure of resistance *in vivo*. While these phenotypic values can be legitimately used to detect non-additive effects, they do not represent a measure of fitness.^39^ In addition to reflecting protection against exposure to the selecting agent, the fitness exhibited by resistant mutants also includes the negative effects that the mutation may have in the absence of drug.^39,40^ Resistance mutations are rarely completely neutral, and (consistent with Fisher’s geometric model of adaptation) the level of protection exhibited by resistant mutants tends to correlate negatively with their fitness.^39^ For measuring fitness, the use of competitive fitness assays is preferable to growth curves because the former integrate all phases of the growth cycle and can capture aspects of competition that are not reflected in single culture experiments.^39^ To this end, we ran a competition assay between the 13 most frequent single mutants used in our experiments and the wild type.

**Figure 2:**
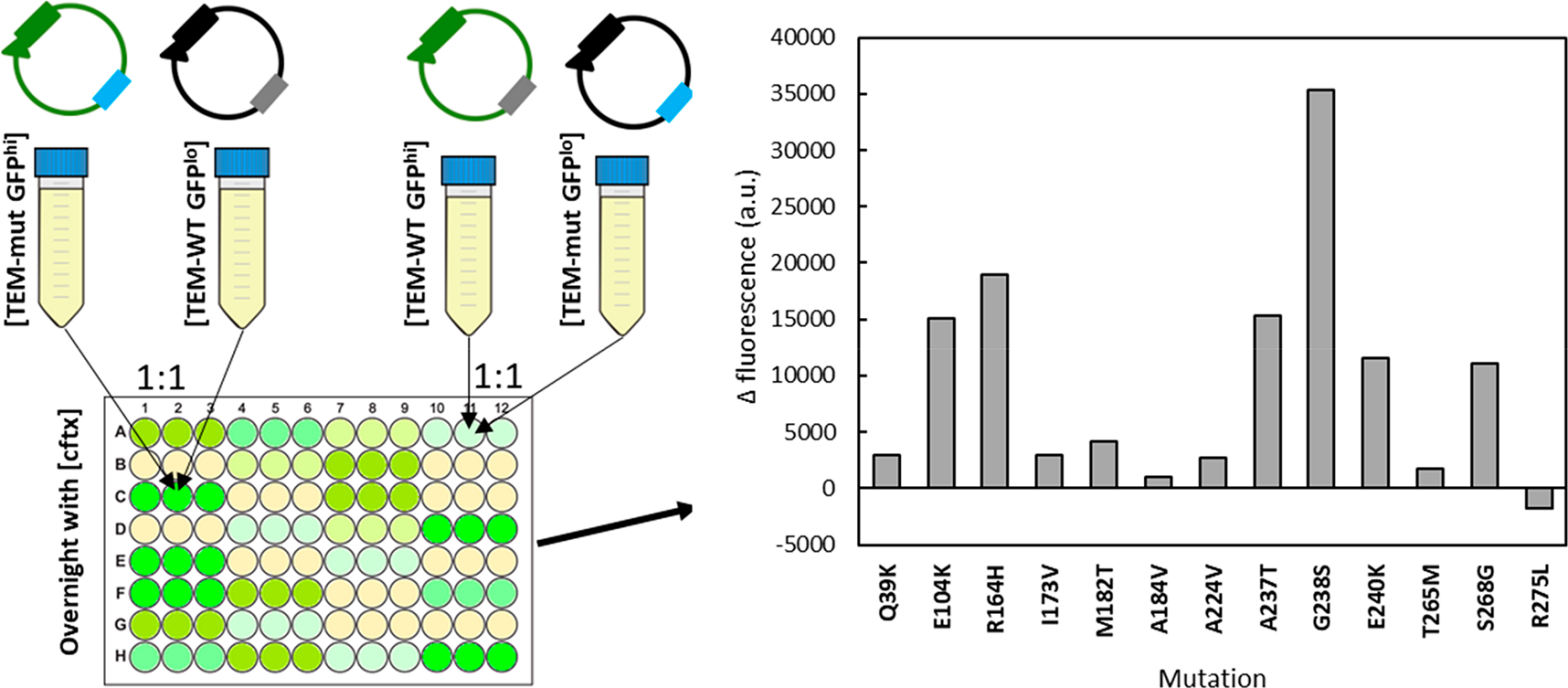
Identification of gain-of-function ESBL mutations using competition assays. [TEM-WT GFP^hi^] and [TEM-mut GFP^lo^] (1:1) co-cultures were grown in 96-well plates in the presence of increasing concentrations of cefotaxime. Similarly, 1:1 [TEM-WT GFP^lo^] and [TEM-mut GFP^hi^] co-cultures were also grown in the presence of cefotaxime. The fluorescence of both competition experiments at the highest concentration of cefotaxime with observable growth was measured. The bar plot shows the difference in average fluorescence signal between the two cultures for each individual mutant.

Briefly, cells transformed with plasmids bearing the original pGFPck plasmid [TEM-WT GFP^hi^] were co-cultured 1:1 with cells transformed with plasmids bearing a version of pGFPck where the Cycle 3 GFP gene bears a point mutation (Q183R) that inactivates fluorescence, [TEM-mut GFP^lo^].^26^ These co-cultures were grown in 96-well plates in the presence of increasing concentrations of cefotaxime and their fluorescence was measured. Similarly, 1:1 co-cultures of [TEM-WT GFP^lo^] and [TEM-mut GFP^hi^] were grown in the presence of cefotaxime and their fluorescence was measured. Figure 2 shows the difference in fluorescence readings between the two co-cultures for each individual mutant for an average of at least three experiments. Measuring the difference between the two co-cultures increases the sensitivity and dynamic range of the assay because, if the difference between TEM-WT and TEM-mut is real, it must go in opposite directions when the reporters are interchanged.

To determine the level of experimental noise, we ran the [TEM-WT GFP^lo^] and [TEM-WT GFP^hi^] 1:1 co-culture 10 times and found the variation range between individual experiments not to exceed 5000 fluorescence units. Mutants E104K, R164H, A237T, G238S, E240K, and S268G resulted in a fluorescence difference well above 5000 units, suggesting that these represent genuine GOF mutations. Indeed, these represent all the known GOF mutations being tested, missing only I173V.^41^ Further, mutations known to have global (M182T) or local (A184V, A224V, T265M) compensatory effects^41,42^ all scored negative.

We find that GOF mutations are highly enriched in epistatic pairs (Table 2). Only 8% of pairs with no GOF mutations exhibit significant epistasis, compared to 59% of pairs with two GOF mutations. When we look at the specific types of epistasis involved, we see massive enrichment of both negative and positive epistasis in mutants with two GOF mutations (0 vs 22.7% and 0 vs 27.3%, respectively). Of particular interest is the representation of reciprocal sign epistatic interactions because of their role driving the ruggedness of the fitness landscape. Pairs with no GOF mutations show no reciprocal sign epistasis, whereas 9.1% of the pairs with two GOF mutations show reciprocal sign epistasis. Similar trends are observed in triple mutants (Table 2). These results are consistent with previous “size effect” observations, that is, with the fact that mutations that produce large phenotypic effects individually also are the main contributors to the ruggedness of fitness landscapes.^43^

**Table 2:** Relationship between Different Categories of Interactions and the Presence of One or Multiple GOF Mutations^a^

no. of mutations	no. of known epistatic pairs observed	no. of GOF mutations	non-significant (%)	positive (%)	negative (%)	reciprocal sign (%)	sign (%)
2	50	0	92.0				8.0
2	106	1	75.5	11.3	1.9	1.9	9.4
2	44	2	40.9	27.3	22.7	9.1	
3	0	0					
3	66	1	63.6	24.2	3.0		
3	102	2	29.4	43.1	7.8	9.8	9.8
3	42	3	14.3	47.6	38.1		

a Results are based on Table S2.

Comparing positive and negative epistatic interactions in Table 2, we observe a preference for positive epistasis (27% vs 23% for doublets, 48% vs 38% for triplets). This bias likely reflects the choice of test mutations, as we selected the ones whose combinations have already undergone significant purifying selection either in nature or in the laboratory.

We also note that, whereas the enrichment for GOF mutations is gradual for positive epistatic interactions (going from 0% for no GOF, to 11% for one GOF, and to 27% for two GOFs), negative and reciprocal sign epistatic interactions are almost completely dependent on the presence of two GOF mutations: 0%, 2%, 23% (negative) and 0%, 2%, 9% (reciprocal sign). Again, the same trend is seen in triplets. This suggests that pairs of negative and reciprocal sign epistatic interactions can be used to identify candidate GOF mutations based on patterns of mutation representation in sequence databases of genes under adaptive selective pressure.

### Study of the Energy of Folding

Enzymes exist in a distribution of different conformations, which can be abstracted as a dynamic equilibrium between a folded and an unfolded state. The energy of folding is the difference in free energy between the protein in its folded and in its unfolded states. For enzymes to act as catalysts, they need a precise molecular configuration, which will generally be associated with their folded state. Indeed, many enzymes can be inactivated by inducing unfolding with a pH or temperature change.

We reasoned that the introduction of mutations in a protein could affect the stability of folding, and thus influence the enzyme activity. Although some experimental methods exist to evaluate folding energies, computational methods allow us to interrogate larger datasets and extract meaningful trends. To estimate the impact of mutations on the enthalpy of folding, here we used FoldX, a molecular-mechanics-based suite for the study of thermodynamic properties of proteins based on an empirical forcefield^44^ (see Methods for details). This computational approach has been shown to capture valid trends across large sets of mutations.^45^

We hypothesized that thermodynamic folding constraints may be relatively constant across a range of protein variants. As a first approximation to exploring the overall thermodynamic landscape of TEM, we performed a virtual alanine scanning of the entire protein. The results, shown in Figure S5, suggest that positions mutated in CCED are enriched for residues with strong interactions with their neighbors. Mutations in these residues would be predicted to affect protein fold and therefore activity, although specific residues actively involved in substrate recognition or catalysis need to be conserved to preserve the protein functionality.

**Figure 3:**
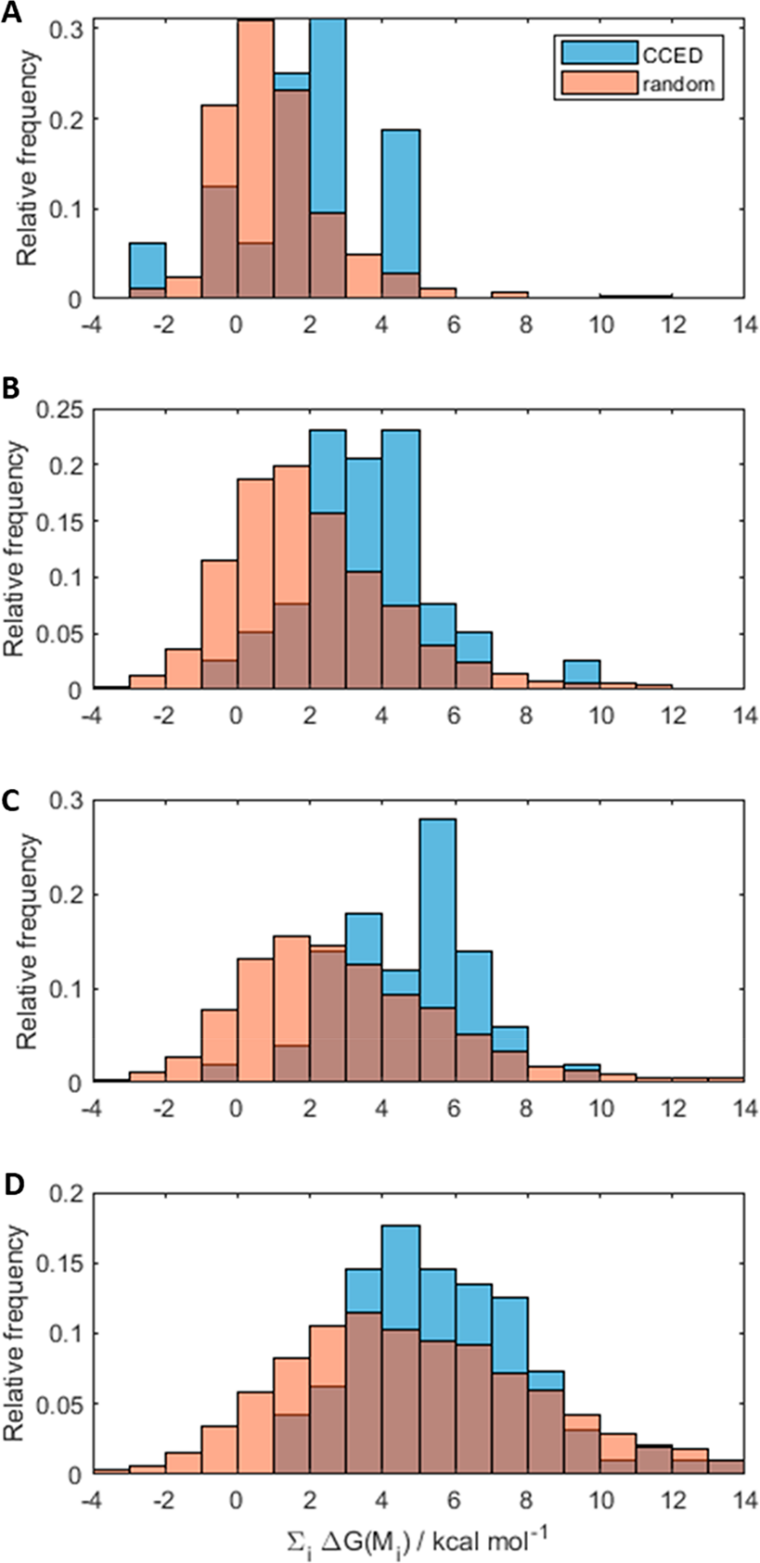
Relative frequency of different CCED mutants containing (A) 1, (B) 2, (C) 3, and (D) >3 mutations and their predicted change in the energy of folding relative to the wild type. Distributions of 1000 random mutants were generated as a baseline comparison (orange) using individual mutations observed in the CCED. In (D), *n* = 4 was used to generate the random distribution. For this analysis, the thermodynamic impact of individual mutations in complex mutants was treated as additive.

Next, we looked at combinations of mutations by considering additive changes in energy after each mutation (Figure 3). This additive use of FoldX does not distort the results significantly. A more refined calculation for the experimentally observed mutants is presented in Figure S6 and agrees with this approximation. An alternative way of looking at this phenomenon is presented in Figure S7, which also suggests that, in CCED mutants, highly destabilizing mutations tend to be accompanied by stabilizing mutations, in agreement with previous suggestions.^14,41,42,46^

We find that the combinations of mutations observed in CCED tend to be more destabilizing than random combinations of mutations drawn from a pool of observed mutations in the same database (see Methods). Importantly, the data also suggest that the energetic destabilization introduced by mutations in TEM-1 is typically limited to the range of 2–8 kcal mol^–1^, irrespective of the number of mutations. This is consistent with the idea that TEM-1 and other proteins have a limited buffer of stability.^37^ In particular, the free energy of folding of TEM-1, Δ*G*_folding_, has been estimated to be around −10.9 kcal mol^–1^ using fluorescence emission spectroscopy and circular dichroism.^47^ Thus, it becomes possible to estimate the cumulative impact that these mutations would have on the fraction of TEM-1 folded at a given temperature (α_f_), given by eq 7, below.

**Figure 4:**
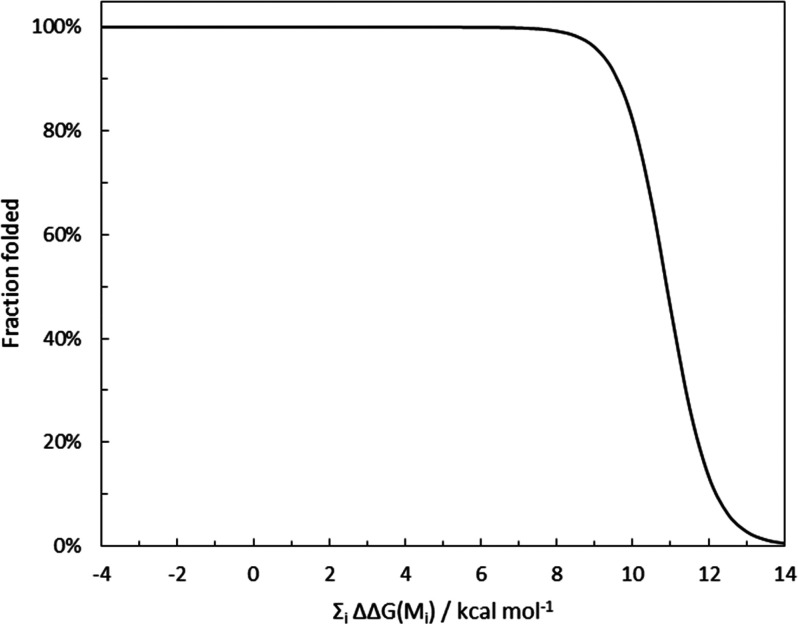
Estimated impact of the change in free energy of folding by mutations in TEM-1 on the fraction of folded protein at 298 K.

Figure 4 shows the estimated impact of the change in free energy of folding induced by mutations in TEM-1 on the fraction of folded protein at 298 K. We can see that the fraction of TEM-1 folded is largely insensitive to mutation-induced changes in its free energy of folding until a threshold of ca. 10 kcal mol^–1^. Our model predicts that mutations that destabilize the protein above this threshold will be greatly disfavored by natural selection because the fraction of folded (active) protein would be dramatically decreased. This analysis shows an excellent agreement with the distributions presented in Figure 3: most mutants observed experimentally have estimated destabilizing effects below 10 kcal mol^–1^, even though, in principle, evolution could explore more destabilizing mutations and combinations of mutations. Our findings also agree with previous reports showing that evolvability requires an excess of enzyme activity relative to the strength of selection, and such enzyme activity may be capped by structural unfolding, among other effects.^48,49^

**Figure 5:**
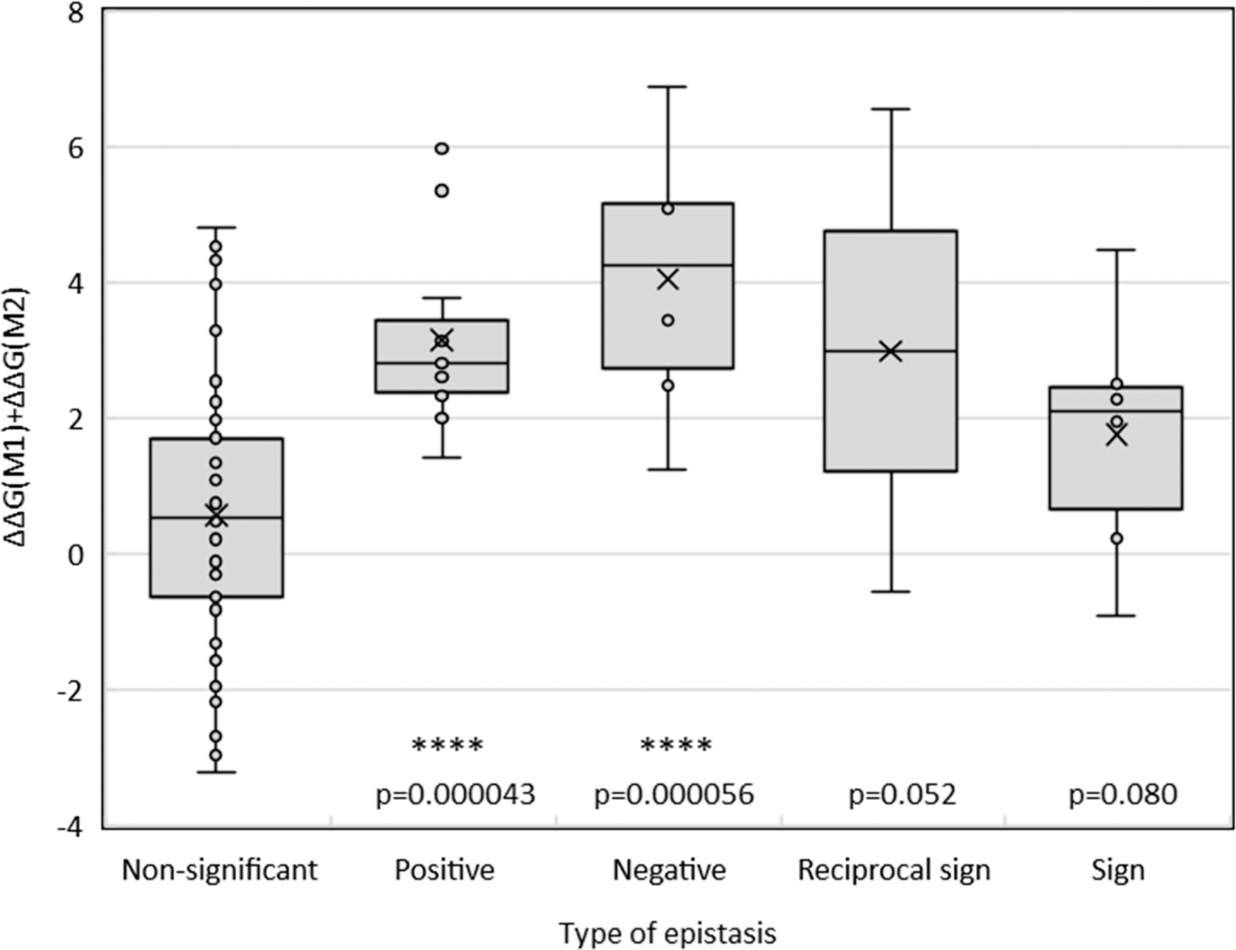
Relationship between the epistasis status of the pairs of mutations included in this study and their estimated effect on the free energy of folding of TEM-1 β-lactamase. The estimated thermodynamic impact of pairs of mutations was modeled using FoldX assuming it considers additivity of effects on free energy of folding (see Methods). The *p*-values indicate the significance of an unpaired single-sided *t*-test between the corresponding type of epistasis and the group with non-significant epistasis.

We next examined the relationship between epistasis and energy of folding (estimating the free energy of folding additively as done above) by looking at the energy of folding for the different categories of functional interactions. The results are shown in Figure 5. We see that the different types of epistatic interactions observed in CCED tend to decrease the folding stability compared to non-epistatic interactions, although with considerable individual variability. Such structural destabilization seems to be particularly pronounced for negative and—surprisingly—positive epistatic pairs of mutations. The generally destabilizing effects of positive epistatic interactions in the context of genetic adaptation are consistent with the idea that more active catalysts against a given substrate tend to be less energetically stable.^50,51^

These results may also explain why negative pairs, which are particularly destabilizing, are only relatively frequent in more complex mutants, as other stabilizing mutations need to be present as well to keep the destabilization below 10 kcal mol^–1^.

### Protein Dynamics and Epistasis

**Figure 6:**
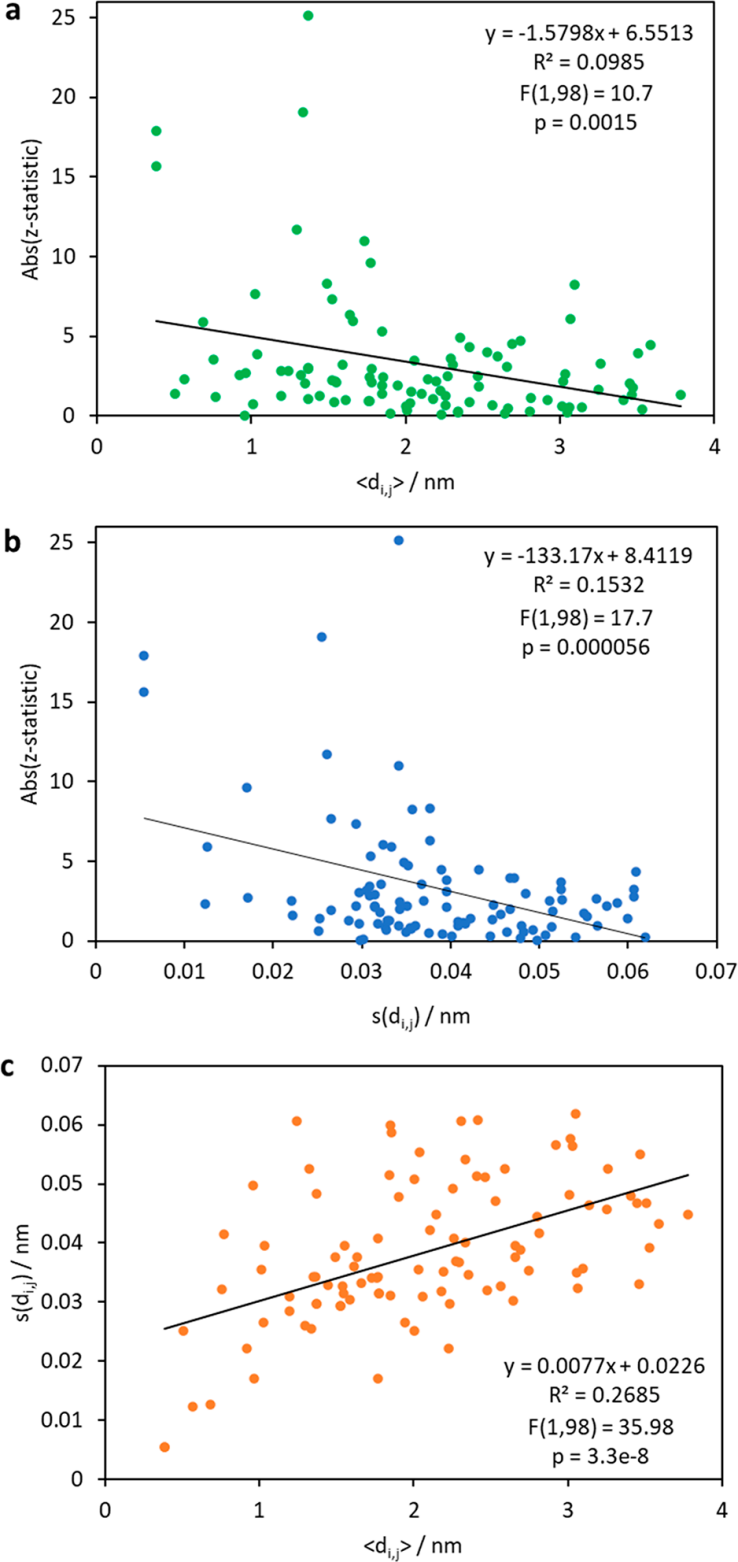
Modeling of epistasis observations based on protein molecular dynamics (MD) parameters. The magnitude of the epistasis between two mutations is quantified here by the absolute value of its *z*-statistic, introduced in this work. Each point in each panel corresponds to a pair of alpha carbons in the protein. For the amino acid positions considered (*i* and *j*), we evaluate their average distance, ⟨*d*_*i,j*_⟩, as well the fluctuation of such distance over time, *s*(*d*_*i,j*_), by carrying out MD simulations of the wild-type structure (PDB: 1ZG4). (a) Relationship between the distance between two residues over time and extent of epistasis. (b) Relationship between the variation of the distance between two residues over time and the extent of epistasis. (c) Relationship between the average distance between two residues and the variability of this distance over time.

Researchers have attempted to study the biophysical determinants of epistatic interactions by investigating the contact of the relevant residues within the 3D protein structure.^13,52^ While some interactions may be explained this way, this approach has limitations, particularly when it comes to long-range interactions.^53^ We hypothesized that, even in the absence of a ligand, protein dynamics alone might help predict epistasis. Figure 6 depicts the magnitude of the epistasis between mutation pairs as measured by the absolute value of its *z*-statistic measured in this work. Each point in the figure corresponds to a pair of residues (alpha carbons) in the protein. We found that strong epistatic interactions tend to arise from positions that are close to each other (quantified by ⟨*d*_*i,j*_⟩), although only a fraction of close residues result in epistatic interactions, which is why the *R*^2^ value is low (Figure 6a). We also observe that strong epistatic interactions tend to correspond to pairs of residues whose movement is coupled (quantified by *s*(*d*_*i,j*_)), although again only a fraction of the pairs included in the analysis exhibit epistasis (Figure 6b). Although these correlations are weak, we find that they are statistically significant. Thus, these parameters that quantify the extent to which two protein residues are structurally and dynamically coupled could help predict if they would interact epistatically or not when mutated. Figure 6c shows that the distance between two residues and the coupling of their movements have a limited correlation, suggesting that they may serve as independent descriptors in predictive models. We also compared other results of epistasis in TEM-1 reported previously (involving E104K or S238S)^34^ and the molecular dynamics (MD) descriptors proposed in this work and detected statistically significant correlations in some cases (Figure S8). Thus, our results suggest that MD-based descriptors might help build predictive models of epistasis along with other types of inputs, such as descriptors of secondary structure, types of mutations involved, and inter-residue contacts.

These results might be extended to the study of protein dynamics in the presence of the ligand. During the catalysis, some GOF positions may exert an important catalytic role and their dynamics may become more strongly coupled to those of other residues. This coupling may explain additional epistatic interactions. Furthermore, it may help explain how changes in one or two residues can impact interactions between many other pairs of residues by altering the catalytic conformation. Indeed, we observe that the fitness landscape can change dramatically with just a few mutations. Moreover, essential residues for enzymatic activity may have an unusually high impact on the folding energy, since highly reactive residues need to be made accessible to the substrate for it to react rapidly. The destabilization in certain parts of the protein may need to be compensated with mutations elsewhere for the protein to remain in the catalytically relevant fold most of the time. Additional factors may also contribute to the observation of epistatic effects, such as the possibility of mutation-induced protein aggregation.

## Conclusions

Understanding the principles governing non-additive (epistatic) functional interactions between mutations selected during genetic adaptation is of high interest because of their enormous impact restricting or opening pathways to increased fitness. Epistasis also provides an important link between structures, properties, and functions of proteins.

In this work, we introduced a quantitative method of assessing epistatic interactions based on growth curves and used it to produce a partial empirical cefotaxime resistance map of combinations of TEM-1 β-lactamase point mutants across positions undergoing adaptive selective pressure. We also generated a library of mutants that evolved under positive selective pressure that we used to guide our approach for exploring the empirical landscape of cefotaxime resistance, to place the results in the context of observed adaptive trajectories, and to model the thermodynamic constraints of genetic adaptation.

Note also that our measurements of epistasis were done based on cefotaxime resistance and not on fitness. Using drug resistance measurements to calculate epistasis is in line with previous analyses of small combinations of point mutations^37^ but not with more recent reports involving more extensive mutant combinations.^34,35^ Measurements of fitness, unlike measurements of resistance, take into account the impact of resistance mutations in the absence of the drug and do not correlate well with MIC measurements.^39^ Nonetheless, 18 out of the 29 pairwise epistatic interactions that we identified were previously reported and only two are inconsistent with previous results, supporting the validity of our analysis (see Table S3).

We find that epistatic pairs are highly enriched GOF mutations, particularly negative and reciprocal sign pairs. This finding implies that GOF phenotypes can be inferred based on the topology of mutation co-occurrence networks, such as the ones constructed for β-lactamases undergoing evolutionary radiation in β-lactamase.^18,27^ We previously observed this correspondence between topology and GOF status in the cases of two class A β-lactamases (TEM-1 and CTX-M-1) and one Class D β-lactamase (OXA-51).^18,27^ This work implies that this is likely a more general principle. GOF mutations are typically the ones that initiate the process of adaptation and that define adaptive trajectories. Their identification is therefore very important to predictive models of drug resistance based on sequencing data, which is important for microorganisms that are slow-growing or hard to culture^54,55^ and for the design of small-molecule inhibitors.

Using the software FoldX to estimate the effects of mutations on TEM-1 folding stability, we find that epistatic pairs tend to be thermodynamically unstable but that the instability of adaptive mutation trajectories is limited by the free energy of folding of the protein. Our results confirm and extend principles previously proposed to underlie adaptive evolution. The idea that thermodynamic stability is not as limiting for genetic adaptation as once thought is in line with a number of recent reports.^11,37,56−58^ Our work thus shows a connection between moderate thermodynamic instability and epistatic interactions.

While thermodynamic stability limits the sequence space accessible to evolution, it does not explain changes in fitness for mutants that do not substantially destabilize the protein. Thus, kinetics factors (dynamics, catalysis) must play an important role. We find evidence that epistasis involves dynamic interactions between residues and introduce and interpret new descriptors based on ligand-free MD simulations that could be useful to predict epistasis, possibly in combination with other descriptors (see, for example, ref (59)). We also hypothesize that related MD descriptors taking into account the ligand and the catalytic process might enable improved predictions of epistasis. This hypothesis is also in agreement with recent results in which epistasis is found to impact the catalytic proficiency of the enzyme without necessarily affecting the free enzyme structure.^60,61^

## Methods

### 1 Approach for Empirical Mapping of Cefotaxime Resistance

To cover a broad sequence space, we decided to include combinations of 17 mutant positions that are found in our CCED database and that include a wide range of frequencies (Figure S1). Based on previous evidence indicating that ESBL TEM-1 variants with alternative substitutions at a given position are largely functionally equivalent,^15^ we selected the amino acid substitution most frequent for each of these positions (listed in Table 1), and in some cases we also included the second most frequent mutation (indicated with an asterisk in Table 1). To measure the impact of higher-order combinations of mutations, we generated 206 mutants containing two or three mutations at these positions (listed in SI03.xlsx).

As a host strain, we used JS200 (*polA*) cells complemented with WT Pol I. This B-strain-based host cell system has increased susceptibility to cefotaxime relative to DH5α or Top10 cells.^18^ To express mutant TEMs we used pGFPck, a plasmid vector that carries kanamycin resistance and Cycle 3 GFP,^26^ and measured growth in the presence of cefotaxime by both OD and fluorescence (see section 3 below). For 13 selected single mutants, gain-of-function activity (seen as protection from cefotaxime toxicity) was determined by measuring fitness using a competition assay (see section 6 below).

### 2 Mutagenesis by the Megaprimer Method

Desired point mutations were introduced into the β-lactamase gene of the vector pGFPuv using the megaprimer method.^62^ Briefly, a 600–800 bp segment of DNA (megaprimer) was generated by polymerase chain reaction (PCR) using gene- and vector-specific primers, one of which contained the relevant mutation. Forward and reverse vector-specific primers were designed to anneal to the pGFPck vector flanking the β-lactamase gene by about 150 bp, amplifying toward the gene. These were also used to sequence final mutant constructs. Gene-specific primers were designed to incorporate the mutation of interest in the middle of the primer, with primer length between 19 and 30 bp. In general, when mutations in the first half of the gene were desired, a forward primer was generated, and it was paired with a reverse vector-specific primer. When mutations in the second half of the gene were desired, a reverse primer was generated, and it was paired with a forward vector-specific primer. When two mutations were within 3 amino acid residues from each other (e.g., M182T_A184V), a gene-specific primer was designed to incorporate both mutations simultaneously. The PCR product was gel purified (Machery-Nagel, cat. no. 740609) and used as a megaprimer to amplify the entire vector (extension time = 2 min/kb) by rolling circle amplification (RCA). Finally, the product from the megaprimer PCR was digested with *DpnI* (New England Biolabs, cat. no. R0176L) to remove template DNA, leaving only newly generated plasmid DNA containing the relevant mutation. This was then transformed into chemically competent Top10 cells (7 μL DNA/50 μL competent cells). Single colonies were used to inoculate liquid overnight cultures, which were then miniprepped (Machery-Nagel, cat. no. 740499), and the plasmid-borne β-lactamase was sequenced. For double mutant constructs, the process was repeated using a single mutant as the template for the first round of PCR, such that the megaprimer contained both desired mutations.

### 3 Quantitative Measurement of Cefotaxime Sensitivity Using Fluorescence

To measure cefotaxime sensitivity, we used deep-well 96-well microtiter plates containing one sterile 3 mm glass bead per well for liquid culture. Overnight cultures were normalized to an OD_600_ = 1, and each well was inoculated with 10 μL in 990 μL LB broth containing kanamycin, chloramphenicol, and cefotaxime. Plates were sealed with Airpore air-permeable sealing strips (Qiagen) and grown overnight at 30 °C with shaking. The following morning, 200 μL of each culture was transferred to a black-walled 96-well microtiter plate (Greiner, cat. no. 655087), and fluorescence (ex: 395 nm, em: 509 nm, autocutoff: 495 nm) and OD_600_ were measured in tandem on a Spectramax M2e plate reader (Molecular Devices).

The range of informative cefotaxime concentrations was wide, from 0.05 μg mL^–1^ to 400 μg mL^–1^. Therefore, we had to customize cefotaxime exposure to each mutant based on an initial set of experiments designed to find the general level of resistance. Based on this information, we performed a second set of experiments, grouping mutants with a similar level of resistance on the same plate, and used this higher resolution data for modeling dose–response curves.

### 4 Modeling of Dose–Response Curves

To identify epistasis (non-additive interactions between the effects of mutations), the following approach was used. The dose–response data for every β-lactamase variant was fit to a classic sigmoidal equation (Hill equation), which is often used for kinetics with inhibition.^63^ The equation has the following form:
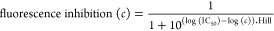
1where log is the decimal logarithm function, *c* is the concentration of antibiotic, and IC_50_ is the concentration that causes 50% inhibition in growth, which is the fitting parameter in the equation. The curves were fit using a Levenberg–Marquardt nonlinear least-squares algorithm as implemented in the MATLAB function *nlinfit*. Both IC_50_ and the Hill slope were allowed to vary in the fitting, and the corresponding variances and covariances were obtained from the fit.

To obtain a more conservative estimate of the standard errors associated with these coefficients, the 155 dose–response curves were re-fit a second time to eq 1, but fixing in this case the Hill coefficient to a value of unity, since under the null hypothesis we would not expect the dose–response curve slope to change. From these regressions, we obtained new estimates for the variance of each log(IC_50_). Since the latter models have one degree of freedom less, the variance estimates tended to be higher. The maximum of both variance estimates was then used to perform the *z*-tests, described below.

### 5 Evaluating the Impact of Mutations Using *z*-Tests

To assess the significance of the effect of individual mutations, we define a *z*-statistic comparing the null hypothesis that the mutation does not affect the IC_50_ (H_0_: IC_50,M_ = IC_50,WT_) vs the alternative hypothesis that it increases it (H_A_: IC_50,M_ > IC_50,WT_):
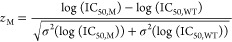
2with the normal distribution taken as the corresponding sampling distribution and the standard errors σ^2^ the maximum of the two fits to each mutant, as indicated above.

The *z*-test was extended to the case of multiple mutations in order to determine the significance of non-additive interactions between mutations (epistasis). The following test was conducted under the null hypothesis that no epistasis is taking place between the mutations M1 and M2 under consideration:
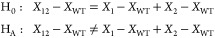
3where *X*_*i*_ = log(IC_50,*i*_).

This leads to the following *z*-statistic:
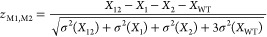
4

The relevant one-sided *z*-test was conducted for every pair of mutations at a 99.95% confidence level. Results are included in the supporting file SI03.xlsx. In frequentist hypothesis testing, decreasing the significance level α of the test reduces the probability of incurring a type I error (erroneously rejecting the null hypothesis, in this case, identifying an epistatic interaction when the mutations have only additive effects). The influence of this decision can be easily evaluated in the supporting file SI04.xlsx.

In the case of triple mutants, we considered all three possible double mutants as baselines on which to compare the effect of an additional mutation. Notice that this can give rise to one mutation being identified as epistatic in the presence of the other two, while other point mutations or mutations in the same triple mutant do not necessarily have to interact epistatically.

If a statistically significant epistasis is detected, then the type of epistasis is assigned according to the first of the following logical rules that evaluates to True:
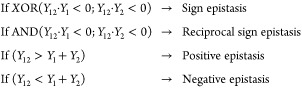
5where *Y*_*i*_ = *X*_*i*_ – *X*_WT_.

### 6 Competition Assays

Our assay is outlined in Figure 1. For each mutant to be tested, we generated a non-fluorescent version by introducing an R at position Q183 of GFP.^26^ For each comparison to be made, the overnight cultures of fluorescent and non-fluorescent transformants were normalized and mixed 50/50. The 50/50 mixture was then used to inoculate 1 mL cultures containing serial dilutions of cefotaxime and grown overnight in a 96-well deep-well plate. The following morning, 200 μL of each culture was transferred into black-walled 96-well microtiter plates, and fluorescence (ex: 395 nm, em: 509 nm, autocutoff: 495 nm) and OD_600_ were measured in tandem on a Spectramax M2e plate reader (Molecular Devices). The highest cefotaxime concentration for which there was observed growth (OD_600_ ≥ 0.1) was used for further analysis.

### 7 Estimating the Effects of Mutations on the Free Energy of Folding

FoldX is a molecular-mechanics-based suite for the study of thermodynamic properties of proteins based on an empirical force field.^44^ The optimization of residues in FoldX is conducted in a sequential manner, one residue at a time. While this is a computationally efficient approach, the absolute accuracy of the results is limited because only a small subspace of conformations for a subset of residues around the mutation in question is considered. This is partly alleviated by applying the same heuristic optimization to both the parent and the mutant enzyme as a pair, which has been shown to inform valid trends across large sets of mutations.^45^ In this work, we applied some modifications of the original approach. Instead of applying the heuristic in FoldX once, we applied the optimization 72 times using a different random seed each time. The number of residues that are optimized across each run of the same mutant is always the same, but the order in which they are optimized and the rotamers selected are not. Crucially, we selected the mutant with the lowest folding free energy (Δ*G*(M*i*)) and the parent enzyme with the lowest free energy (Δ*G*(WT)) among the 72 pairs of structures and then subtracted their energies to assess the impact of the mutation on the stability of the enzyme (ΔΔ*G*(M*i*) = Δ*G*(M*i*) – Δ*G*(WT)). Notice that the parent and mutant thus selected may come from different FoldX output pairs. Because a relatively large number of runs are conducted, the structures selected should be a better approximation of the lowest energy configuration for both the mutant and the parent than those obtained from a single heuristic run. Moreover, this approach makes FoldX’s output more consistent irrespective of the initial seed used. On the downside, this is a much more computationally expensive approach than the original FoldX pipeline. Thus, we parallelized its execution in the Hummingbird cluster at UCSC (each node comprising an Intel Xeon E5-2650v4, 2×12 cores, 128 GB DRAM). The 64-bit version of FoldX 5.0 for Linux was used. A high-quality structure of TEM-1 was obtained from the PDB (1ZG4, 1.55 Å resolution, *R*_free_ = 0.240, *R*_work_ = 0.185). An initial optimization of the parent structure was carried out using the RepairPDB command before further manipulation. Special options used with BuildModel in FoldX include “--vdwDesign=2 --out-pdb=false --nrotamers=5”. The free energy values computed by FoldX were used without further transformation. Secondary structure assignments were obtained from the PDB structure using the software STRIDE.^64^

### 8 Relationship between Free Energy of Folding and Fraction of Folded Protein

Mutations can affect the free energy of folding (Δ*G*_folding_) and, consequently, the fraction of TEM-1 folded at a given temperature:
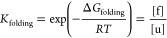
6where *K*_folding_ is the folding equilibrium constant and [f] and [u] are the activities (concentrations) of the protein in the folded and unfolded states. Rearranging this expression and introducing the fraction of folded protein, α_f_:
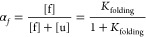
7

### 9 Molecular Dynamics Simulations

MD simulations were carried out using Acellera’s SimpleRun.^65^ The crystal structure of TEM-1 β-lactamase (PDB: 1zg4) was prepared using SystemBuilder, after removing water molecules from the structure. The system was protonated at a pH of 7.4, and 13 608 water molecules were incorporated along with the protein in a cubic box. Equilibration is done in the NPT ensemble (300 K, 1 atm). First, 500 steps of minimization are run. Then simulation starts, and 1 kcal mol^–1^ restraints are applied throughout the first half of the equilibration run on CA atoms and ligands, and 0.1 kcal mol^–1^ on other heavy atoms. In the second part of the equilibration, the restraints are scaled down linearly over time to 0 kcal mol^–1^. The equilibration run spans a total of 10 ns. Then, a 15 ns production is carried out in NVT with no restraints. Simulations were carried out at 300 K using a 4 fs integration step. The production run trajectory was analyzed using the Python library *mdtraj*.^66^ Custom scripts were created to compute the average pairwise distance between CA of all residues during the run. We also computed the standard deviation of each pairwise distance during the run.

## Supporting Information Available

Mutations in CCED database (Figure S1), dose–response fitting (Figure S2), IC_50_ for all variants (Table S1), epistatic interactions identified (Table S2), previously reported interactions (Table S3), structural model of TEM-1 (Figure S3), relationship between ampicillin and cefotaxime epistasis (Figure S4), virtual alanine scanning (Figure S5), FoldX results (Figure S6), destabilizing effects in CCED (Figure S7), and molecular dynamics results (Figure S8) (PDF)
SI01.xlsx, CCED raw data (XLSX)
SI02.xlsx, experimental raw data (XLSX)
SI03.xlsx, epistatic analysis of experimental data (XLSX)
SI04.xlsx, epistatic analysis of CCED data (XLSX)
