# Supplementary material for: Experimental and In Silico Analysis of TEM β-Lactamase Adaptive Evolution
Source: ACS Infect Dis. 2022 Nov 15;8(12):2451–63. doi: 10.1021/acsinfecdis.2c00216 (PMC9745794; doi:10.1021/acsinfecdis.2c00216)
Supplement: Supplementary file 1 — id2c00216_si_001.pdf [file id2c00216_si_001.pdf]

**SUPPORTING INFORMATION**

# Experimental and *in silico* analysis of TEM β-lactamase adaptive evolution

Melissa Standley[1,*], Vincent Blay[1,2,*,**], Violeta Beleva Guthrie[3], Jay Kim[1], Audrey Lyman[4], Andrés Moya[2,5,6], Rachel Karchin[3], Manel Camps[1,**]

[1]Department of Microbiology and Environmental Toxicology, University of California, Santa Cruz, Santa Cruz, CA 95064, USA.

[2]Institute for Integrative Systems Biology (I[2]SysBio), Universitat de València and Spanish Research Council (CSIC), 46980 Valencia, Spain.

[3]Department of Biomedical Engineering and Institute for Computational Medicine, The Johns Hopkins University, Baltimore, MD, 21218, USA.

[4]Dovetail Genomics, LLC. 100 Enterprise Way, Scotts Valley, CA 95066, USA.

[5]Foundation for the Promotion of Sanitary and Biomedical Research of Valencian Community (FISABIO), 46021 Valencia, Spain.

[6]CIBER in Epidemiology and Public Health (CIBEResp), 28029 Madrid, Spain.

* These authors contributed equally and are first co-authors.

** Co-corresponding authors: vroger@ucsc.edu (V.B.), mcamps@ucsc.edu (M.C.).

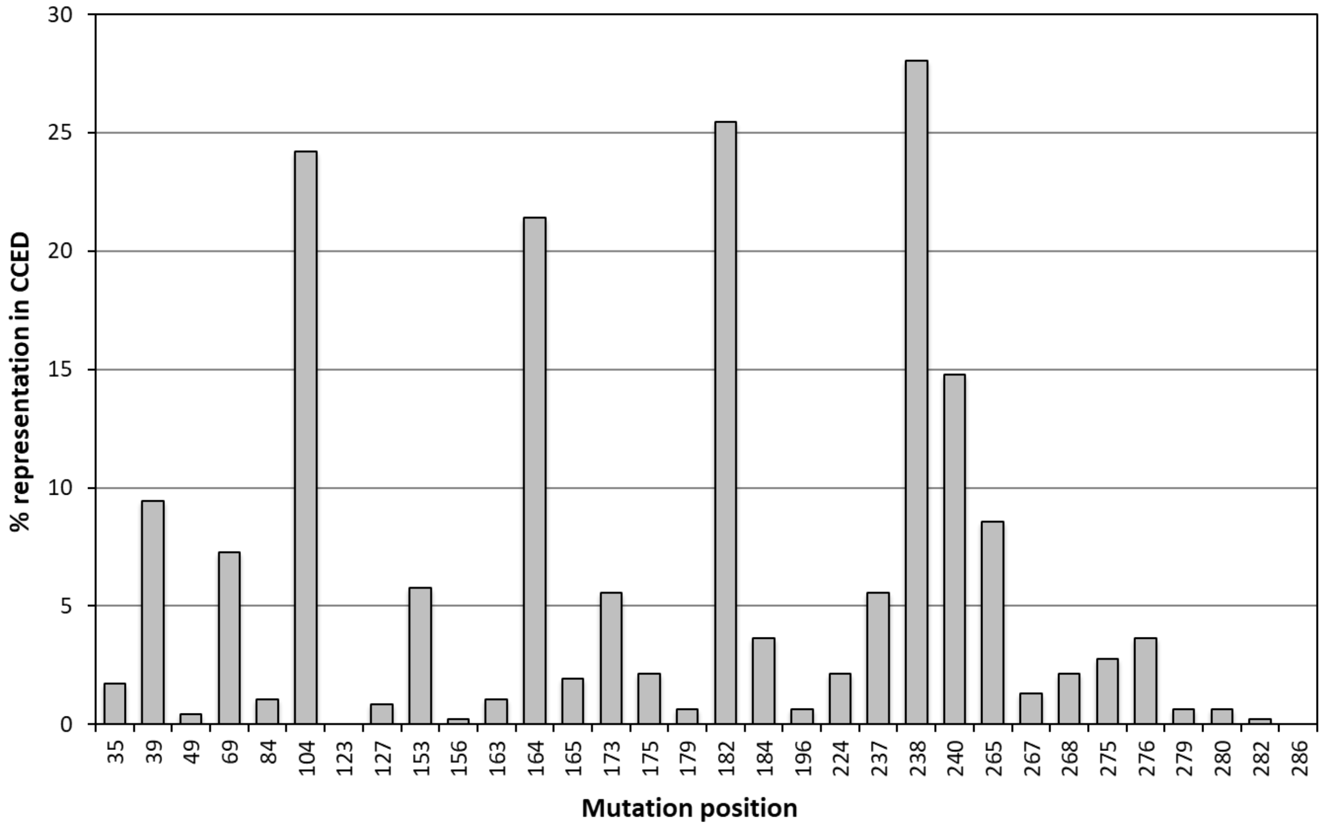


**Figure S1.** Representation of various point mutations in the combined clinical and experimental CCED database, shown as a percentage of sequences containing the mutation.

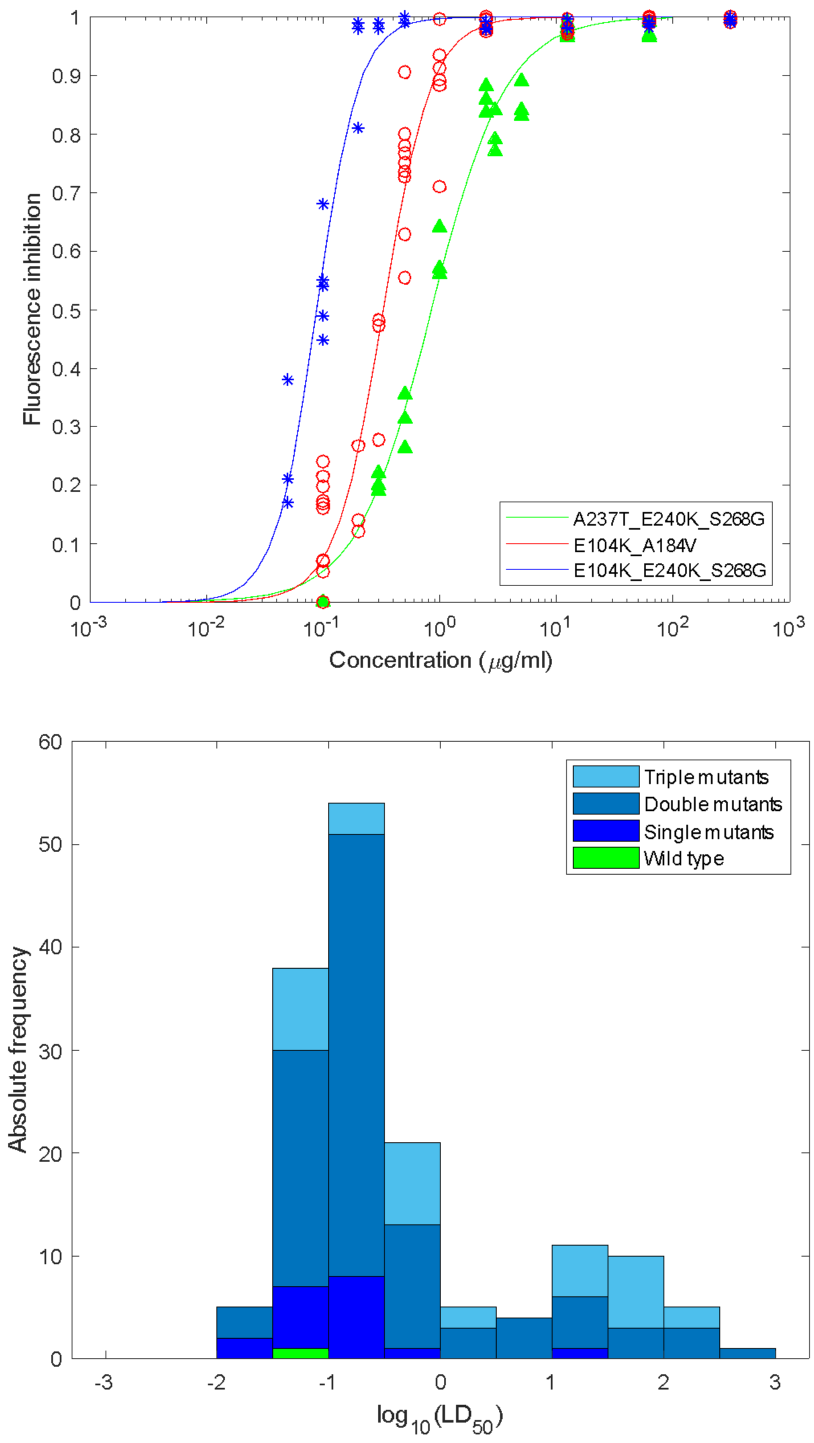


**Figure S2**. a) Example of fitting the two-parameter dose-response curve (2-parameter Eq. 1) to three different mutants, and b) distribution of the fitted $IC_{50}$ for the different β-lactamase variants in this study.

**Table S1.** Point estimates and 99% confidence intervals of the $IC_{50}$ for the 155 enzyme variants in this study. $IC_{50}$ values were obtained by non-linear regression of the data to Eq. 1 (2-parameter version), and the fold change sorted results for the wild type. LB and UB denote lower and upper boundaries of the confidence intervals, respectively. The variances used to compute the confidence intervals are the largest obtained in the data regression to the 1-parameter and 2-parameter versions of Eq. 1.

| Variant | $\log_{10}(IC_{50})$ | $IC_{50}$ (μg/ml) | LB 99% CI $\log_{10}(IC_{50})$ | UB 99% CI $\log_{10}(IC_{50})$ | LB 99% CI $IC_{50}$ (μg/ml) | UB 99% CI $IC_{50}$ (μg/ml) | $\log_{10}(IC_{50})$ $-\log_{10}(IC_{50})_{WT}$ |
|---|---|---|---|---|---|---|---|
| M182T_G238S | 2.78 | 6.03E+02 | 2.33 | 3.23 | 2.16E+02 | 1.68E+03 | 3.90 |
| G238S_T265M | 2.26 | 1.80E+02 | 2.11 | 2.40 | 1.29E+02 | 2.53E+02 | 3.38 |
| R164H_A237T_E240K | 2.24 | 1.74E+02 | 1.96 | 2.52 | 9.19E+01 | 3.30E+02 | 3.36 |
| G238S_E240K | 2.20 | 1.57E+02 | 2.03 | 2.36 | 1.08E+02 | 2.30E+02 | 3.32 |
| E104K_R164H_M182T | 2.12 | 1.33E+02 | 1.97 | 2.28 | 9.24E+01 | 1.90E+02 | 3.24 |
| E104K_G238S | 2.05 | 1.12E+02 | 1.91 | 2.19 | 8.21E+01 | 1.54E+02 | 3.17 |
| R164H_A184V_E240K | 1.96 | 9.03E+01 | 1.76 | 2.16 | 5.70E+01 | 1.43E+02 | 3.08 |
| R164H_I173V_E240K | 1.91 | 8.11E+01 | 1.82 | 2.00 | 6.54E+01 | 1.01E+02 | 3.03 |
| R164H_A224V_E240K | 1.83 | 6.73E+01 | 1.74 | 1.92 | 5.49E+01 | 8.25E+01 | 2.95 |
| A184V_G238S | 1.79 | 6.14E+01 | 1.66 | 1.92 | 4.55E+01 | 8.29E+01 | 2.91 |
| E104K_R164H_A237T | 1.75 | 5.68E+01 | 1.64 | 1.87 | 4.36E+01 | 7.39E+01 | 2.87 |
| G238S_R275L | 1.67 | 4.70E+01 | 1.47 | 1.88 | 2.93E+01 | 7.54E+01 | 2.79 |
| E104K_R164H_I173V | 1.66 | 4.58E+01 | 1.46 | 1.86 | 2.89E+01 | 7.26E+01 | 2.78 |
| Q39K_G238S | 1.65 | 4.52E+01 | 1.39 | 1.92 | 2.46E+01 | 8.29E+01 | 2.78 |
| R164H_I173V_A184V | 1.52 | 3.35E+01 | 1.39 | 1.66 | 2.43E+01 | 4.61E+01 | 2.65 |
| E104K_R164H_A224V | 1.52 | 3.32E+01 | 1.35 | 1.69 | 2.24E+01 | 4.90E+01 | 2.64 |
| I173V_G238S | 1.40 | 2.51E+01 | 1.10 | 1.70 | 1.26E+01 | 5.01E+01 | 2.52 |
| A224V_G238S | 1.34 | 2.20E+01 | 1.10 | 1.59 | 1.25E+01 | 3.89E+01 | 2.46 |
| G238S | 1.32 | 2.11E+01 | 1.21 | 1.43 | 1.64E+01 | 2.72E+01 | 2.44 |
| R164H_M182T_A224V | 1.27 | 1.86E+01 | 1.08 | 1.46 | 1.19E+01 | 2.88E+01 | 2.39 |
| E104K_R164H_A184V | 1.26 | 1.81E+01 | 1.11 | 1.41 | 1.29E+01 | 2.56E+01 | 2.38 |
| R164H_M182T_T265M | 1.23 | 1.71E+01 | 1.09 | 1.38 | 1.22E+01 | 2.40E+01 | 2.35 |
| R164H_A184V_A237T | 1.14 | 1.39E+01 | 0.99 | 1.30 | 9.76E+00 | 1.98E+01 | 2.26 |
| R164H_I173V | 1.09 | 1.24E+01 | 0.96 | 1.23 | 9.11E+00 | 1.69E+01 | 2.21 |
| Q39K_E104K_M182T | 1.03 | 1.08E+01 | 0.95 | 1.12 | 8.92E+00 | 1.30E+01 | 2.15 |
| L21F_G238S | 1.02 | 1.04E+01 | 0.73 | 1.30 | 5.36E+00 | 2.02E+01 | 2.14 |
| R164H_E240K | 1.00 | 1.01E+01 | 0.87 | 1.13 | 7.47E+00 | 1.36E+01 | 2.12 |
| R164H_M182T | 0.94 | 8.62E+00 | 0.79 | 1.08 | 6.20E+00 | 1.20E+01 | 2.06 |
| E104K_R164H | 0.87 | 7.45E+00 | 0.80 | 0.95 | 6.27E+00 | 8.85E+00 | 1.99 |
| R164S_A237T | 0.73 | 5.34E+00 | -0.01 | 1.46 | 9.77E-01 | 2.92E+01 | 1.85 |
| G238S_D254G | 0.52 | 3.28E+00 | 0.28 | 0.75 | 1.89E+00 | 5.68E+00 | 1.64 |
| R164H_A224V_S268G | 0.50 | 3.13E+00 | 0.38 | 0.61 | 2.40E+00 | 4.08E+00 | 1.62 |
| R164H_A237T | 0.49 | 3.09E+00 | 0.41 | 0.57 | 2.55E+00 | 3.74E+00 | 1.61 |
| E104K_M182T_A184V | 0.22 | 1.66E+00 | 0.15 | 0.28 | 1.43E+00 | 1.92E+00 | 1.34 |
| E104K_M182T | 0.06 | 1.14E+00 | -0.04 | 0.15 | 9.12E-01 | 1.42E+00 | 1.18 |
| Q39K_R164H | 0.04 | 1.09E+00 | -0.19 | 0.26 | 6.46E-01 | 1.84E+00 | 1.16 |
| R164H_A224V | -0.02 | 9.64E-01 | -0.20 | 0.17 | 6.27E-01 | 1.48E+00 | 1.10 |
| A237T_E240K_S268G | -0.07 | 8.51E-01 | -0.15 | 0.01 | 7.04E-01 | 1.03E+00 | 1.05 |
| R164H_A184V | -0.10 | 8.04E-01 | -0.23 | 0.04 | 5.82E-01 | 1.11E+00 | 1.03 |
| E104K_T265M_S268G | -0.11 | 7.77E-01 | -0.24 | 0.02 | 5.78E-01 | 1.04E+00 | 1.01 |
| E104K_A237T_S268G | -0.11 | 7.71E-01 | -0.23 | 0.01 | 5.86E-01 | 1.02E+00 | 1.01 |
| R164H_A224V_T265M | -0.14 | 7.27E-01 | -0.24 | -0.03 | 5.70E-01 | 9.28E-01 | 0.98 |
| E104K_S268G | -0.16 | 6.85E-01 | -0.28 | -0.05 | 5.30E-01 | 8.85E-01 | 0.96 |
| R164H_T265M | -0.19 | 6.51E-01 | -0.33 | -0.05 | 4.72E-01 | 8.98E-01 | 0.93 |
| E104K_T265M | -0.20 | 6.28E-01 | -0.34 | -0.06 | 4.53E-01 | 8.70E-01 | 0.92 |
| R164H_S268G | -0.27 | 5.43E-01 | -0.46 | -0.07 | 3.44E-01 | 8.56E-01 | 0.86 |
| E104K_R164H_E240K | -0.32 | 4.82E-01 | -0.45 | -0.18 | 3.51E-01 | 6.62E-01 | 0.80 |
| R164H | -0.38 | 4.16E-01 | -0.50 | -0.26 | 3.14E-01 | 5.52E-01 | 0.74 |
| A224V_E240K | -0.41 | 3.90E-01 | -0.63 | -0.19 | 2.37E-01 | 6.41E-01 | 0.71 |
| R164H_G238S | -0.41 | 3.88E-01 | -0.67 | -0.15 | 2.15E-01 | 7.00E-01 | 0.71 |
| E104K_I173V | -0.43 | 3.72E-01 | -0.56 | -0.30 | 2.73E-01 | 5.07E-01 | 0.69 |
| E240K_S268G | -0.43 | 3.70E-01 | -0.57 | -0.30 | 2.71E-01 | 5.05E-01 | 0.69 |
| A237T_G238S | -0.44 | 3.66E-01 | -0.62 | -0.25 | 2.38E-01 | 5.62E-01 | 0.68 |
| E104K_I173V_A224V | -0.46 | 3.46E-01 | -0.61 | -0.32 | 2.47E-01 | 4.84E-01 | 0.66 |

| | | | | | | | |
|---|---|---|---|---|---|---|---|
| E104K_I173V_A237T | -0.46 | 3.44E-01 | -0.66 | -0.27 | 2.21E-01 | 5.37E-01 | 0.66 |
| E104K_A184V_A224V | -0.48 | 3.30E-01 | -0.63 | -0.33 | 2.32E-01 | 4.69E-01 | 0.64 |
| E104K_A184V | -0.48 | 3.29E-01 | -0.59 | -0.37 | 2.55E-01 | 4.24E-01 | 0.64 |
| E104K_M182T_E240K | -0.52 | 2.99E-01 | -0.67 | -0.37 | 2.12E-01 | 4.22E-01 | 0.60 |
| M182T_A237T_E240K | -0.57 | 2.70E-01 | -0.69 | -0.45 | 2.04E-01 | 3.56E-01 | 0.55 |
| I173V_E240K | -0.58 | 2.63E-01 | -0.72 | -0.44 | 1.89E-01 | 3.64E-01 | 0.54 |
| E240K_T265M | -0.61 | 2.48E-01 | -0.84 | -0.37 | 1.43E-01 | 4.29E-01 | 0.52 |
| E104K | -0.65 | 2.22E-01 | -0.76 | -0.55 | 1.74E-01 | 2.84E-01 | 0.47 |
| H153R_T265M | -0.66 | 2.17E-01 | -0.97 | -0.36 | 1.07E-01 | 4.40E-01 | 0.46 |
| E104K_E240K | -0.68 | 2.09E-01 | -0.82 | -0.54 | 1.53E-01 | 2.87E-01 | 0.44 |
| H153R_I173V | -0.71 | 1.95E-01 | -0.92 | -0.50 | 1.19E-01 | 3.20E-01 | 0.41 |
| R164H_D254G | -0.73 | 1.88E-01 | -1.07 | -0.38 | 8.42E-02 | 4.19E-01 | 0.39 |
| H153R_E240K | -0.75 | 1.77E-01 | -1.12 | -0.38 | 7.54E-02 | 4.16E-01 | 0.37 |
| Q39K_H153R | -0.76 | 1.72E-01 | -0.99 | -0.54 | 1.03E-01 | 2.86E-01 | 0.36 |
| M182T_A237T | -0.77 | 1.71E-01 | -0.90 | -0.63 | 1.26E-01 | 2.32E-01 | 0.35 |
| A184V_E240K | -0.77 | 1.70E-01 | -0.94 | -0.60 | 1.16E-01 | 2.50E-01 | 0.35 |
| Q39K_A184V | -0.78 | 1.66E-01 | -1.01 | -0.55 | 9.70E-02 | 2.84E-01 | 0.34 |
| E104K_A237T | -0.82 | 1.52E-01 | -1.03 | -0.60 | 9.26E-02 | 2.50E-01 | 0.30 |
| H153R_S268G | -0.82 | 1.51E-01 | -1.06 | -0.58 | 8.68E-02 | 2.61E-01 | 0.30 |
| D254G | -0.83 | 1.49E-01 | -1.02 | -0.64 | 9.60E-02 | 2.30E-01 | 0.29 |
| I173V_D254G | -0.83 | 1.48E-01 | -1.08 | -0.58 | 8.25E-02 | 2.65E-01 | 0.29 |
| I173V_T265M | -0.83 | 1.47E-01 | -1.08 | -0.58 | 8.27E-02 | 2.61E-01 | 0.29 |
| H153R_M182T | -0.84 | 1.46E-01 | -1.10 | -0.58 | 7.97E-02 | 2.66E-01 | 0.28 |
| L21F_E104K | -0.85 | 1.41E-01 | -1.16 | -0.54 | 6.87E-02 | 2.89E-01 | 0.27 |
| M182T | -0.85 | 1.40E-01 | -0.98 | -0.72 | 1.04E-01 | 1.89E-01 | 0.27 |
| A184V_S268G | -0.86 | 1.39E-01 | -1.12 | -0.60 | 7.65E-02 | 2.53E-01 | 0.26 |
| E104K_A224V | -0.86 | 1.37E-01 | -1.05 | -0.68 | 8.99E-02 | 2.08E-01 | 0.26 |
| M182T_E240K | -0.87 | 1.33E-01 | -0.97 | -0.78 | 1.07E-01 | 1.66E-01 | 0.25 |
| A237G_G238S | -0.89 | 1.30E-01 | -1.14 | -0.64 | 7.28E-02 | 2.30E-01 | 0.23 |
| E240K | -0.89 | 1.27E-01 | -1.02 | -0.77 | 9.56E-02 | 1.70E-01 | 0.23 |
| Q39K_E104K | -0.90 | 1.26E-01 | -1.01 | -0.79 | 9.68E-02 | 1.64E-01 | 0.22 |
| L21F_I173V | -0.92 | 1.21E-01 | -1.13 | -0.71 | 7.47E-02 | 1.95E-01 | 0.20 |
| D254G_T265M | -0.92 | 1.20E-01 | -1.23 | -0.60 | 5.83E-02 | 2.49E-01 | 0.20 |
| I173V_M182T | -0.93 | 1.17E-01 | -1.13 | -0.73 | 7.36E-02 | 1.86E-01 | 0.19 |
| H153R | -0.93 | 1.17E-01 | -1.09 | -0.77 | 8.06E-02 | 1.69E-01 | 0.19 |
| A237T | -0.93 | 1.16E-01 | -1.03 | -0.84 | 9.37E-02 | 1.44E-01 | 0.19 |
| I173V | -0.94 | 1.16E-01 | -1.08 | -0.79 | 8.29E-02 | 1.62E-01 | 0.18 |
| H153R_A184V | -0.94 | 1.15E-01 | -1.23 | -0.65 | 5.91E-02 | 2.24E-01 | 0.18 |
| L21F_S268G | -0.95 | 1.13E-01 | -1.23 | -0.66 | 5.90E-02 | 2.18E-01 | 0.18 |
| A224V_S268G | -0.95 | 1.12E-01 | -1.08 | -0.82 | 8.28E-02 | 1.53E-01 | 0.17 |
| L21F_E240K | -0.97 | 1.08E-01 | -1.29 | -0.65 | 5.16E-02 | 2.25E-01 | 0.15 |
| I173V_A184V | -0.97 | 1.06E-01 | -1.14 | -0.81 | 7.27E-02 | 1.55E-01 | 0.15 |
| E104K_I173V_E240K | -0.97 | 1.06E-01 | -1.17 | -0.78 | 6.76E-02 | 1.66E-01 | 0.15 |
| M182T_D254G | -0.98 | 1.06E-01 | -1.21 | -0.75 | 6.22E-02 | 1.79E-01 | 0.14 |
| I173V_A237T | -0.98 | 1.05E-01 | -1.15 | -0.81 | 7.09E-02 | 1.57E-01 | 0.14 |
| A184V_T265M | -0.98 | 1.05E-01 | -1.26 | -0.70 | 5.51E-02 | 2.01E-01 | 0.14 |
| M182T_A184V | -0.98 | 1.05E-01 | -1.12 | -0.84 | 7.57E-02 | 1.46E-01 | 0.14 |
| A224V_T265M | -0.98 | 1.05E-01 | -1.11 | -0.85 | 7.84E-02 | 1.40E-01 | 0.14 |
| A237T_S268G | -0.98 | 1.04E-01 | -1.12 | -0.85 | 7.63E-02 | 1.41E-01 | 0.14 |
| I173V_A224V | -0.99 | 1.04E-01 | -1.15 | -0.82 | 7.03E-02 | 1.52E-01 | 0.14 |
| H153R_D254G | -0.99 | 1.03E-01 | -1.18 | -0.80 | 6.67E-02 | 1.60E-01 | 0.14 |
| A237T_E240K | -0.99 | 1.02E-01 | -1.13 | -0.85 | 7.33E-02 | 1.43E-01 | 0.13 |
| A224V_A237T | -0.99 | 1.01E-01 | -1.26 | -0.73 | 5.55E-02 | 1.85E-01 | 0.13 |
| A184V_A237T | -0.99 | 1.01E-01 | -1.10 | -0.89 | 7.89E-02 | 1.30E-01 | 0.13 |
| A237G | -1.00 | 1.01E-01 | -1.23 | -0.76 | 5.83E-02 | 1.75E-01 | 0.13 |
| I173V_S268G | -1.00 | 1.01E-01 | -1.22 | -0.77 | 6.00E-02 | 1.69E-01 | 0.12 |
| A237T_T265M | -1.00 | 1.00E-01 | -1.28 | -0.72 | 5.31E-02 | 1.90E-01 | 0.12 |
| A184V | -1.00 | 9.97E-02 | -1.11 | -0.90 | 7.84E-02 | 1.27E-01 | 0.12 |
| E104K_N175I_A184V | -1.00 | 9.96E-02 | -1.21 | -0.80 | 6.23E-02 | 1.59E-01 | 0.12 |
| E104K_A237T_E240K | -1.00 | 9.89E-02 | -1.22 | -0.79 | 6.06E-02 | 1.62E-01 | 0.12 |
| Q39K_S268G | -1.01 | 9.81E-02 | -1.31 | -0.71 | 4.93E-02 | 1.95E-01 | 0.11 |
| T265M | -1.01 | 9.70E-02 | -1.15 | -0.88 | 7.09E-02 | 1.33E-01 | 0.11 |
| M182T_A184V_A237T | -1.02 | 9.54E-02 | -1.21 | -0.83 | 6.17E-02 | 1.48E-01 | 0.10 |
| E104K_E240K_S268G | -1.05 | 8.83E-02 | -1.20 | -0.90 | 6.27E-02 | 1.25E-01 | 0.07 |
| A184V_A224V | -1.06 | 8.80E-02 | -1.21 | -0.91 | 6.23E-02 | 1.24E-01 | 0.06 |

| | | | | | | | |
|---|---|---|---|---|---|---|---|
| D254G_S268G | -1.06 | 8.76E-02 | -1.33 | -0.78 | 4.66E-02 | 1.65E-01 | 0.06 |
| L21F_H153R | -1.06 | 8.69E-02 | -1.28 | -0.85 | 5.29E-02 | 1.43E-01 | 0.06 |
| S268G | -1.06 | 8.67E-02 | -1.18 | -0.94 | 6.60E-02 | 1.14E-01 | 0.06 |
| Q39K_A237T | -1.07 | 8.47E-02 | -1.21 | -0.94 | 6.23E-02 | 1.15E-01 | 0.05 |
| Q39K_M182T | -1.08 | 8.37E-02 | -1.25 | -0.91 | 5.67E-02 | 1.24E-01 | 0.04 |
| Q39K_D254G | -1.08 | 8.25E-02 | -1.39 | -0.77 | 4.05E-02 | 1.68E-01 | 0.04 |
| E104K_N175I | -1.08 | 8.22E-02 | -1.19 | -0.98 | 6.52E-02 | 1.04E-01 | 0.04 |
| Q39K_T265M | -1.10 | 7.86E-02 | -1.36 | -0.84 | 4.32E-02 | 1.43E-01 | 0.02 |
| L21F_A237T | -1.11 | 7.75E-02 | -1.29 | -0.93 | 5.09E-02 | 1.18E-01 | 0.01 |
| N175I | -1.11 | 7.68E-02 | -1.33 | -0.89 | 4.63E-02 | 1.28E-01 | 0.01 |
| WT | -1.12 | 7.58E-02 | -1.22 | -1.02 | 6.05E-02 | 9.49E-02 | 0.00 |
| Q39K_I173V | -1.12 | 7.57E-02 | -1.37 | -0.87 | 4.27E-02 | 1.34E-01 | 0.00 |
| Q39K_E240K | -1.13 | 7.49E-02 | -1.29 | -0.96 | 5.13E-02 | 1.09E-01 | 0.00 |
| Q39K_A237G | -1.14 | 7.25E-02 | -1.47 | -0.81 | 3.42E-02 | 1.54E-01 | -0.02 |
| L21F_A184V | -1.16 | 6.91E-02 | -1.32 | -1.00 | 4.81E-02 | 9.94E-02 | -0.04 |
| A184V_D254G | -1.17 | 6.77E-02 | -1.39 | -0.95 | 4.10E-02 | 1.12E-01 | -0.05 |
| L21F | -1.18 | 6.67E-02 | -1.34 | -1.01 | 4.59E-02 | 9.68E-02 | -0.06 |
| A224V_T265M_S268G | -1.18 | 6.59E-02 | -1.39 | -0.97 | 4.04E-02 | 1.08E-01 | -0.06 |
| N175I_A184V | -1.18 | 6.54E-02 | -1.40 | -0.97 | 3.97E-02 | 1.08E-01 | -0.06 |
| Q39K_E104K_A237T | -1.20 | 6.35E-02 | -1.37 | -1.02 | 4.24E-02 | 9.50E-02 | -0.08 |
| A224V | -1.20 | 6.30E-02 | -1.43 | -0.97 | 3.74E-02 | 1.06E-01 | -0.08 |
| H153R_A224V | -1.25 | 5.59E-02 | -1.43 | -1.08 | 3.71E-02 | 8.41E-02 | -0.13 |
| M182T_S268G | -1.25 | 5.56E-02 | -1.68 | -0.83 | 2.11E-02 | 1.46E-01 | -0.13 |
| A237T_D254G | -1.26 | 5.48E-02 | -1.50 | -1.02 | 3.18E-02 | 9.45E-02 | -0.14 |
| E104K_N175I_E240K | -1.32 | 4.75E-02 | -1.55 | -1.10 | 2.84E-02 | 7.96E-02 | -0.20 |
| G238S_S268G | -1.33 | 4.67E-02 | -1.50 | -1.17 | 3.19E-02 | 6.83E-02 | -0.21 |
| A237G_S268G | -1.34 | 4.59E-02 | -1.49 | -1.18 | 3.20E-02 | 6.59E-02 | -0.22 |
| M182T_A224V | -1.37 | 4.25E-02 | -1.72 | -1.02 | 1.91E-02 | 9.46E-02 | -0.25 |
| Q39K_A237T_E240K | -1.43 | 3.70E-02 | -1.59 | -1.27 | 2.56E-02 | 5.33E-02 | -0.31 |
| Q39K_A224V | -1.44 | 3.66E-02 | -1.65 | -1.22 | 2.24E-02 | 5.96E-02 | -0.32 |
| M182T_T265M | -1.51 | 3.09E-02 | -2.10 | -0.92 | 8.01E-03 | 1.19E-01 | -0.39 |
| R275L | -1.59 | 2.58E-02 | -1.82 | -1.35 | 1.51E-02 | 4.42E-02 | -0.47 |
| Q39K | -1.68 | 2.08E-02 | -1.98 | -1.38 | 1.05E-02 | 4.14E-02 | -0.56 |
| N175I_E240K | -1.80 | 1.57E-02 | -2.63 | -0.98 | 2.36E-03 | 1.04E-01 | -0.68 |
| T265M_S268G | -1.86 | 1.39E-02 | -2.76 | -0.95 | 1.73E-03 | 1.12E-01 | -0.74 |
| R164S | -4.73 | 1.86E-05 | -12.26 | 2.80 | 5.52E-13 | 6.27E+02 | -3.61 |

**Table S2.** Epistatic interactions identified according to the *z*-test defined in Eq. 4. Results are sorted by *p*-value, for $p < 0.0005$. For reference, $IC_{50,WT}$ = 0.076 μg/ml.

| Variant | $IC_{50}$ (μg/ml) | Mutation1 | Mutation2 | $IC_{50}$ M1 (μg/ml) | $IC_{50}$ M2 (μg/ml) | z-statistic | p-value | Type of epistasis |
|---|---|---|---|---|---|---|---|---|
| G238S_S268G | 4.67E-02 | G238S | S268G | 2.11E+01 | 8.67E-02 | -25.16 | 0.00000 | **Reciprocal sign epistasis** |
| R164H_G238S | 3.88E-01 | R164H | G238S | 4.16E-01 | 2.11E+01 | -19.08 | 0.00000 | **Negative epistasis** |
| A237T_G238S | 3.66E-01 | A237T | G238S | 1.16E-01 | 2.11E+01 | -17.89 | 0.00000 | **Negative epistasis** |
| R164H_A237T_E240K | 1.74E+02 | A237T_E240K | R164H | 1.02E-01 | 4.16E-01 | 17.74 | 0.00000 | **Positive epistasis** |
| Q39K_E104K_M182T | 1.08E+01 | Q39K_E104K | M182T | 1.26E-01 | 1.40E-01 | 17.10 | 0.00000 | **Positive epistasis** |
| R164H_I173V_E240K | 8.11E+01 | I173V_E240K | R164H | 2.63E-01 | 4.16E-01 | 17.08 | 0.00000 | **Positive epistasis** |
| E104K_R164H_E240K | 4.82E-01 | R164H_E240K | E104K | 1.01E+01 | 2.22E-01 | -17.07 | 0.00000 | **Negative epistasis** |
| R164H_A184V_E240K | 9.03E+01 | A184V_E240K | R164H | 1.70E-01 | 4.16E-01 | 15.89 | 0.00000 | **Positive epistasis** |
| Q39K_E104K_M182T | 1.08E+01 | Q39K_M182T | E104K | 8.37E-02 | 2.22E-01 | 15.76 | 0.00000 | **Positive epistasis** |
| A237G_G238S | 1.30E-01 | A237G | G238S | 1.01E-01 | 2.11E+01 | -15.64 | 0.00000 | **Negative epistasis** |
| R164H_A184V_E240K | 9.03E+01 | R164H_A184V | E240K | 8.04E-01 | 1.27E-01 | 15.08 | 0.00000 | **Positive epistasis** |
| E104K_R164H_A237T | 5.68E+01 | E104K_A237T | R164H | 1.52E-01 | 4.16E-01 | 14.99 | 0.00000 | **Positive epistasis** |
| R164H_A224V_E240K | 6.73E+01 | R164H_A224V | E240K | 9.64E-01 | 1.27E-01 | 14.49 | 0.00000 | **Positive epistasis** |
| E104K_R164H_E240K | 4.82E-01 | E104K_R164H | E240K | 7.45E+00 | 1.27E-01 | -14.23 | 0.00000 | **Negative epistasis** |
| E104K_R164H_A224V | 3.32E+01 | E104K_A224V | R164H | 1.37E-01 | 4.16E-01 | 13.32 | 0.00000 | **Positive epistasis** |
| R164H_A184V_A237T | 1.39E+01 | A184V_A237T | R164H | 1.01E-01 | 4.16E-01 | 13.14 | 0.00000 | **Positive epistasis** |
| R164H_I173V_A184V | 3.35E+01 | R164H_A184V | I173V | 8.04E-01 | 1.16E-01 | 12.72 | 0.00000 | **Positive epistasis** |
| R164H_A224V_E240K | 6.73E+01 | A224V_E240K | R164H | 3.90E-01 | 4.16E-01 | 12.68 | 0.00000 | **Positive epistasis** |
| E104K_R164H_M182T | 1.33E+02 | E104K_M182T | R164H | 1.14E+00 | 4.16E-01 | 12.52 | 0.00000 | **Positive epistasis** |
| R164H_I173V_A184V | 3.35E+01 | I173V_E240K | R164H | 2.63E-01 | 4.16E-01 | 12.50 | 0.00000 | **Positive epistasis** |
| R164H_I173V | 1.24E+01 | R164H | I173V | 4.16E-01 | 1.16E-01 | 11.72 | 0.00000 | **Positive epistasis** |
| R164H_A237T_E240K | 1.74E+02 | R164H_A237T | E240K | 3.09E+00 | 1.27E-01 | 11.44 | 0.00000 | **Positive epistasis** |
| R164H_M182T_A224V | 1.86E+01 | M182T_A224V | R164H | 4.25E-02 | 4.16E-01 | 11.34 | 0.00000 | **Sign epistasis** |
| E104K_R164H_I173V | 4.58E+01 | E104K_I173V | R164H | 3.72E-01 | 4.16E-01 | 11.26 | 0.00000 | **Positive epistasis** |
| Q39K_E104K_M182T | 1.08E+01 | E104K_M182T | Q39K | 1.14E+00 | 2.08E-02 | 11.04 | 0.00000 | **Sign epistasis** |
| R164H_E240K | 1.01E+01 | R164H | E240K | 4.16E-01 | 1.27E-01 | 10.98 | 0.00000 | **Positive epistasis** |
| E104K_E240K_S268G | 8.83E-02 | E104K_S268G | E240K | 6.85E-01 | 1.27E-01 | -10.49 | 0.00000 | **Negative epistasis** |
| R164H_M182T_T265M | 1.71E+01 | R164H_T265M | M182T | 6.51E-01 | 1.40E-01 | 10.35 | 0.00000 | **Positive epistasis** |
| E104K_E240K_S268G | 8.83E-02 | E240K_S268G | E104K | 3.70E-01 | 2.22E-01 | -10.14 | 0.00000 | **Negative epistasis** |
| R164H_A184V_A237T | 1.39E+01 | R164H_A184V | A237T | 8.04E-01 | 1.16E-01 | 9.79 | 0.00000 | **Positive epistasis** |
| R164H_M182T | 8.62E+00 | R164H | M182T | 4.16E-01 | 1.40E-01 | 9.63 | 0.00000 | **Positive epistasis** |
| E104K_R164H_A184V | 1.81E+01 | E104K_A184V | R164H | 3.29E-01 | 4.16E-01 | 9.47 | 0.00000 | **Positive epistasis** |
| E104K_R164H_M182T | 1.33E+02 | E104K_R164H | M182T | 7.45E+00 | 1.40E-01 | 9.39 | 0.00000 | **Positive epistasis** |
| E104K_R164H_A224V | 3.32E+01 | R164H_A224V | E104K | 9.64E-01 | 2.22E-01 | 8.75 | 0.00000 | **Positive epistasis** |
| A237T_E240K_S268G | 8.51E-01 | A237T_E240K | S268G | 1.02E-01 | 8.67E-02 | 8.52 | 0.00000 | **Positive epistasis** |
| E104K_R164H_A237T | 5.68E+01 | R164H_A237T | E104K | 3.09E+00 | 2.22E-01 | 8.51 | 0.00000 | **Positive epistasis** |
| R164H_M182T_T265M | 1.71E+01 | M182T_T265M | R164H | 3.09E-02 | 4.16E-01 | 8.39 | 0.00000 | **Sign epistasis** |
| E104K_R164H | 7.45E+00 | E104K | R164H | 2.22E-01 | 4.16E-01 | 8.30 | 0.00000 | **Positive epistasis** |
| G238S_D254G | 3.28E+00 | G238S | D254G | 2.11E+01 | 1.49E-01 | -8.24 | 0.00000 | **Negative epistasis** |
| E104K_R164H_A184V | 1.81E+01 | R164H_A184V | E104K | 8.04E-01 | 2.22E-01 | 8.17 | 0.00000 | **Positive epistasis** |
| R164H_M182T_A224V | 1.86E+01 | R164H_A224V | M182T | 9.64E-01 | 1.40E-01 | 7.91 | 0.00000 | **Positive epistasis** |
| R164H_A237T_E240K | 1.74E+02 | R164H_E240K | A237T | 1.01E+01 | 1.16E-01 | 7.77 | 0.00000 | **Positive epistasis** |
| E104K_M182T_E240K | 2.99E-01 | E104K_M182T | E240K | 1.14E+00 | 1.27E-01 | -7.74 | 0.00000 | **Negative epistasis** |
| E104K_R164H_A237T | 5.68E+01 | E104K_R164H | A237T | 7.45E+00 | 1.16E-01 | 7.68 | 0.00000 | **Positive epistasis** |
| G238S_T265M | 1.80E+02 | G238S | T265M | 2.11E+01 | 9.70E-02 | 7.66 | 0.00000 | **Positive epistasis** |
| E104K_M182T_A184V | 1.66E+00 | M182T_A184V | E104K | 1.05E-01 | 2.22E-01 | 7.53 | 0.00000 | **Positive epistasis** |
| R164H_A224V_E240K | 6.73E+01 | R164H_E240K | A224V | 1.01E+01 | 6.30E-02 | 7.40 | 0.00000 | **Sign epistasis** |
| R164H_A237T | 3.09E+00 | R164H | A237T | 4.16E-01 | 1.16E-01 | 7.32 | 0.00000 | **Positive epistasis** |
| E104K_I173V_E240K | 1.06E-01 | I173V_E240K | E104K | 2.63E-01 | 2.22E-01 | -7.28 | 0.00000 | **Negative epistasis** |
| R164H_A224V_S268G | 3.13E+00 | A224V_T265M | R164H | 1.05E-01 | 4.16E-01 | 7.20 | 0.00000 | **Positive epistasis** |
| R164H_A184V_E240K | 9.03E+01 | R164H_E240K | A184V | 1.01E+01 | 9.97E-02 | 7.13 | 0.00000 | **Positive epistasis** |
| A237T_E240K_S268G | 8.51E-01 | A237T_S268G | E240K | 1.04E-01 | 1.27E-01 | 6.93 | 0.00000 | **Positive epistasis** |
| R164H_I173V_E240K | 8.11E+01 | R164H_E240K | I173V | 1.01E+01 | 1.16E-01 | 6.90 | 0.00000 | **Positive epistasis** |
| E104K_R164H_M182T | 1.33E+02 | R164H_M182T | E104K | 8.62E+00 | 2.22E-01 | 6.46 | 0.00000 | **Positive epistasis** |
| E104K_I173V_E240K | 1.06E-01 | E104K_I173V | E240K | 3.72E-01 | 1.27E-01 | -6.46 | 0.00000 | **Negative epistasis** |
| M182T_G238S | 6.03E+02 | M182T | G238S | 1.40E-01 | 2.11E+01 | 6.32 | 0.00000 | **Positive epistasis** |
| R164H_A224V_S268G | 3.13E+00 | R164H_S268G | A224V | 5.43E-01 | 6.30E-02 | 6.15 | 0.00000 | **Sign epistasis** |
| Q39K_R164H | 1.09E+00 | Q39K | R164H | 2.08E-02 | 4.16E-01 | 6.06 | 0.00000 | **Sign epistasis** |

| | | | | | | | | |
|---|---|---|---|---|---|---|---|---|
| G238S_R275L | 4.70E+01 | G238S | R275L | 2.11E+01 | 2.58E-02 | 5.93 | 0.00000 | **Sign epistasis** |
| G238S_E240K | 1.57E+02 | G238S | E240K | 2.11E+01 | 1.27E-01 | 5.92 | 0.00000 | **Positive epistasis** |
| R164H_I173V_E240K | 8.11E+01 | R164H_I173V | E240K | 1.24E+01 | 1.27E-01 | 5.81 | 0.00000 | **Positive epistasis** |
| E104K_R164H_A224V | 3.32E+01 | E104K_R164H | A224V | 7.45E+00 | 6.30E-02 | 5.70 | 0.00000 | **Sign epistasis** |
| R164H_A184V_A237T | 1.39E+01 | R164H_A237T | A184V | 3.09E+00 | 9.97E-02 | 5.33 | 0.00000 | **Positive epistasis** |
| E104K_A237T_S268G | 7.71E-01 | E104K_A237T | S268G | 1.52E-01 | 8.67E-02 | 5.29 | 0.00000 | **Positive epistasis** |
| Q39K_G238S | 4.52E+01 | Q39K | G238S | 2.08E-02 | 2.11E+01 | 5.29 | 0.00000 | **Sign epistasis** |
| Q39K_E104K_A237T | 6.35E-02 | Q39K_A237T | E104K | 8.47E-02 | 2.22E-01 | -5.29 | 0.00000 | **Reciprocal sign epistasis** |
| Q39K_A237T_E240K | 3.70E-02 | Q39K_A237T | E240K | 8.47E-02 | 1.27E-01 | -5.27 | 0.00000 | **Reciprocal sign epistasis** |
| E104K_R164H_I173V | 4.58E+01 | E104K_R164H | I173V | 7.45E+00 | 1.16E-01 | 5.20 | 0.00000 | **Positive epistasis** |
| Q39K_A184V | 1.66E-01 | Q39K | A184V | 2.08E-02 | 9.97E-02 | 4.93 | 0.00000 | **Sign epistasis** |
| A237T_D254G | 5.48E-02 | A237T | D254G | 1.16E-01 | 1.49E-01 | -4.71 | 0.00000 | **Reciprocal sign epistasis** |
| E104K_M182T_A184V | 1.66E+00 | E104K_A184V | M182T | 3.29E-01 | 1.40E-01 | 4.59 | 0.00000 | **Positive epistasis** |
| E104K_N175I_E240K | 4.75E-02 | E104K_E240K | N175I | 2.09E-01 | 7.68E-02 | -4.57 | 0.00000 | **Reciprocal sign epistasis** |
| Q39K_E104K_A237T | 6.35E-02 | Q39K_E104K | A237T | 1.26E-01 | 1.16E-01 | -4.50 | 0.00000 | **Reciprocal sign epistasis** |
| E104K_M182T | 1.14E+00 | E104K | M182T | 2.22E-01 | 1.40E-01 | 4.48 | 0.00000 | **Positive epistasis** |
| Q39K_H153R | 1.72E-01 | Q39K | H153R | 2.08E-02 | 1.17E-01 | 4.47 | 0.00000 | **Sign epistasis** |
| Q39K_A237T_E240K | 3.70E-02 | Q39K_E240K | A237T | 7.49E-02 | 1.16E-01 | -4.38 | 0.00001 | **Sign epistasis** |
| E104K_S268G | 6.85E-01 | E104K | S268G | 2.22E-01 | 8.67E-02 | 4.34 | 0.00001 | **Positive epistasis** |
| E104K_A237T_E240K | 9.89E-02 | E104K_E240K | A237T | 2.09E-01 | 1.16E-01 | -4.27 | 0.00001 | **Negative epistasis** |
| A224V_E240K | 3.90E-01 | A224V | E240K | 6.30E-02 | 1.27E-01 | 3.98 | 0.00003 | **Sign epistasis** |
| E104K_A237T_S268G | 7.71E-01 | A237T_S268G | E104K | 1.04E-01 | 2.22E-01 | 3.98 | 0.00004 | **Positive epistasis** |
| R164H_A224V_S268G | 3.13E+00 | R164H_A224V | S268G | 9.64E-01 | 8.67E-02 | 3.96 | 0.00004 | **Positive epistasis** |
| R164H_D254G | 1.88E-01 | R164H | D254G | 4.16E-01 | 1.49E-01 | -3.96 | 0.00004 | **Negative epistasis** |
| E104K_E240K_S268G | 8.83E-02 | E104K_E240K | S268G | 2.09E-01 | 8.67E-02 | -3.95 | 0.00004 | **Negative epistasis** |
| E104K_A237T_E240K | 9.89E-02 | A237T_E240K | E104K | 1.02E-01 | 2.22E-01 | -3.93 | 0.00004 | **Negative epistasis** |
| E104K_N175I_A184V | 9.96E-02 | E104K_A184V | N175I | 3.29E-01 | 7.68E-02 | -3.90 | 0.00005 | **Negative epistasis** |
| E104K_I173V_E240K | 1.06E-01 | E104K_E240K | I173V | 2.09E-01 | 1.16E-01 | -3.90 | 0.00005 | **Negative epistasis** |
| E240K_S268G | 3.70E-01 | E240K | S268G | 1.27E-01 | 8.67E-02 | 3.83 | 0.00006 | **Positive epistasis** |
| E104K_N175I_E240K | 4.75E-02 | E104K_N175I | E240K | 8.22E-02 | 1.27E-01 | -3.83 | 0.00006 | **Reciprocal sign epistasis** |
| E104K_N175I | 8.22E-02 | E104K | N175I | 2.22E-01 | 7.68E-02 | -3.71 | 0.00010 | **Negative epistasis** |
| E104K_T265M_S268G | 7.77E-01 | T265M_S268G | E104K | 1.39E-02 | 2.22E-01 | 3.63 | 0.00014 | **Sign epistasis** |
| A184V_D254G | 6.77E-02 | A184V | D254G | 9.97E-02 | 1.49E-01 | -3.58 | 0.00017 | **Reciprocal sign epistasis** |
| Q39K_S268G | 9.81E-02 | Q39K | S268G | 2.08E-02 | 8.67E-02 | 3.55 | 0.00019 | **Sign epistasis** |
| E104K_R164H_E240K | 4.82E-01 | E104K_E240K | R164H | 2.09E-01 | 4.16E-01 | -3.48 | 0.00025 | **Negative epistasis** |
| A184V_G238S | 6.14E+01 | A184V | G238S | 9.97E-02 | 2.11E+01 | 3.46 | 0.00027 | **Positive epistasis** |

**Table S3.** Concordance between our experimental analysis of epistasis and reports of epistatic or likely epistatic interactions in the literature.

| M1 | M2 | z-statistic | p-value | Epistasis | Literature** | Concordance | Refs. |
|---|---|---|---|---|---|---|---|
| G238S | E104K | 2.55 | p=0.005 | - | positive | yes* | (1-5) |
| G238S | R164H | -19.8 | p<0005 | negative | negative | yes | (6,7) |
| G238S | M182T | 6.3 | p<0005 | positive | positive | yes | (4,5) |
| G238S | A237G | -15.6 | p<0005 | negative | negative | yes | (7) |
| G238S | A237T | -17.9 | p<0005 | negative | negative | yes | (7) |
| G238S | E240K | 5.91 | p<0005 | positive | positive | yes | (8-10) |
| E104K | R164H | 8.30 | p<0005 | positive | positive | yes | (11) |
| E104K | M182T | 4.48 | p<0005 | positive | positive | yes | (5) |
| E104K | G238S | 2.55 | p=0.005 | - | positive | yes* | (2-5) |
| E104K | E240K | -2.41 | p=0.008 | - | negative | yes* | (7) |
| E104K | S268G | 4.34 | p<0005 | positive | negative | no | (12) |
| R164H | A237T | 7.30 | p<0005 | positive | positive | yes | (13) |
| R164H | I173V | 11.72 | p<0005 | positive | positive | yes | (14) |
| R164H | E240K | 10.98 | p<0005 | positive | positive | yes | (1,11,13,15) |
| R164H | Q39K | 6.06 | p<0005 | sign | positive | yes | (10,16) |
| A184V | M182T | -2.30 | p=0.010 | - | negative | yes* | (7) |
| M182T | T265M | -3.20 | p<0.0005 | negative | negative | yes | (7) |
| A237T | E240K | -2.71 | p=0.003 | - | positive | no | (13) |

* Trend in the same direction, with significance 0.0005<p<0.05.

** Evidence can include consistent co-selection in directed or natural evolution.

References Table S3


S1. Barlow, M. and Hall, B.G. (2002) Predicting evolutionary potential: in vitro evolution accurately reproduces natural evolution of the tem beta-lactamase. *Genetics*, **160**, 823-832.

S2. Stemmer, W.P. (1994) Rapid evolution of a protein in vitro by DNA shuffling. *Nature*, **370**, 389-391.

S3. Viadiu, H., Osuna, J., Fink, A.L. and Soberon, X. (1995) A new TEM beta-lactamase double mutant with broadened specificity reveals substrate-dependent functional interactions. *J Biol Chem*, **270**, 781-787.

S4. Wang, X., Minasov, G. and Shoichet, B.K. (2002) Evolution of an antibiotic resistance enzyme constrained by stability and activity trade-offs. *J Mol Biol*, **320**, 85-95.

S5. Weinreich, D.M., Delaney, N.F., Depristo, M.A. and Hartl, D.L. (2006) Darwinian evolution can follow only very few mutational paths to fitter proteins. *Science*, **312**, 111-114.

S6. Dellus-Gur, E., Elias, M., Caselli, E., Prati, F., Salverda, M.L., de Visser, J.A., Fraser, J.S. and Tawfik, D.S. (2015) Negative Epistasis and Evolvability in TEM-1 beta-Lactamase--The Thin Line between an Enzyme's Conformational Freedom and Disorder. *J Mol Biol*, **427**, 2396-2409.

S7. Salverda, M.L., Dellus, E., Gorter, F.A., Debets, A.J., van der Oost, J., Hoekstra, R.F., Tawfik, D.S. and de Visser, J.A. (2011) Initial mutations direct alternative pathways of protein evolution. *PLoS Genet*, **7**, e1001321.

S8. De Wals, P.Y., Doucet, N. and Pelletier, J.N. (2009) High tolerance to simultaneous active-site mutations in TEM-1 beta-lactamase: Distinct mutational paths provide more generalized beta-lactam recognition. *Protein Sci*, **18**, 147-160.

S9. Venkatachalam, K.V., Huang, W., LaRocco, M. and Palzkill, T. (1994) Characterization of TEM-1 beta-lactamase mutants from positions 238 to 241 with increased catalytic efficiency for ceftazidime. *J Biol Chem*, **269**, 23444-23450.

S10. Crona, K., Patterson, D., Stack, K., Greene, D., Goulart, C., Mahmudi, M., Jacobs, S.D., Kallman, M. and Barlow, M. (2013) Antibiotic resistance landscapes: a quantification of theory-data incompatibility for fitness landscapes. *arXiv:1303.3842v1 [q-bio.PE]*

S11. Sowek, J.A., Singer, S.B., Ohringer, S., Malley, M.F., Dougherty, T.J., Gougoutas, J.Z. and Bush, K. (1991) Substitution of lysine at position 104 or 240 of TEM-1pTZ18R beta-lactamase enhances the effect of serine-164 substitution on hydrolysis or affinity for cephalosporins and the monobactam aztreonam. *Biochemistry*, **30**, 3179-3188.

S12. Vakulenko, S. and Golemi, D. (2002) Mutant TEM beta-lactamase producing resistance to ceftazidime, ampicillins, and beta-lactamase inhibitors. *Antimicrob Agents Chemother*, **46**, 646-653.

S13. Blazquez, J., Negri, M.C., Morosini, M.I., Gomez-Gomez, J.M. and Baquero, F. (1998) A237T as a modulating mutation in naturally occurring extended-spectrum TEM-type beta-lactamases. *Antimicrob Agents Chemother*, **42**, 1042-1044.

S14. Barlow, M. and Hall, B.G. (2003) Experimental prediction of the natural evolution of antibiotic resistance. *Genetics*, **163**, 1237-1241.

S15. Raquet, X., Vanhove, M., Lamotte-Brasseur, J., Goussard, S., Courvalin, P. and Frere, J.M. (1995) Stability of TEM beta-lactamase mutants hydrolyzing third generation cephalosporins. *Proteins*, **23**, 63-72.

S16. Jacoby, G.A. and Medeiros, A.A. (1991) More extended-spectrum beta-lactamases. *Antimicrob Agents Chemother*, **35**, 1697-1704.

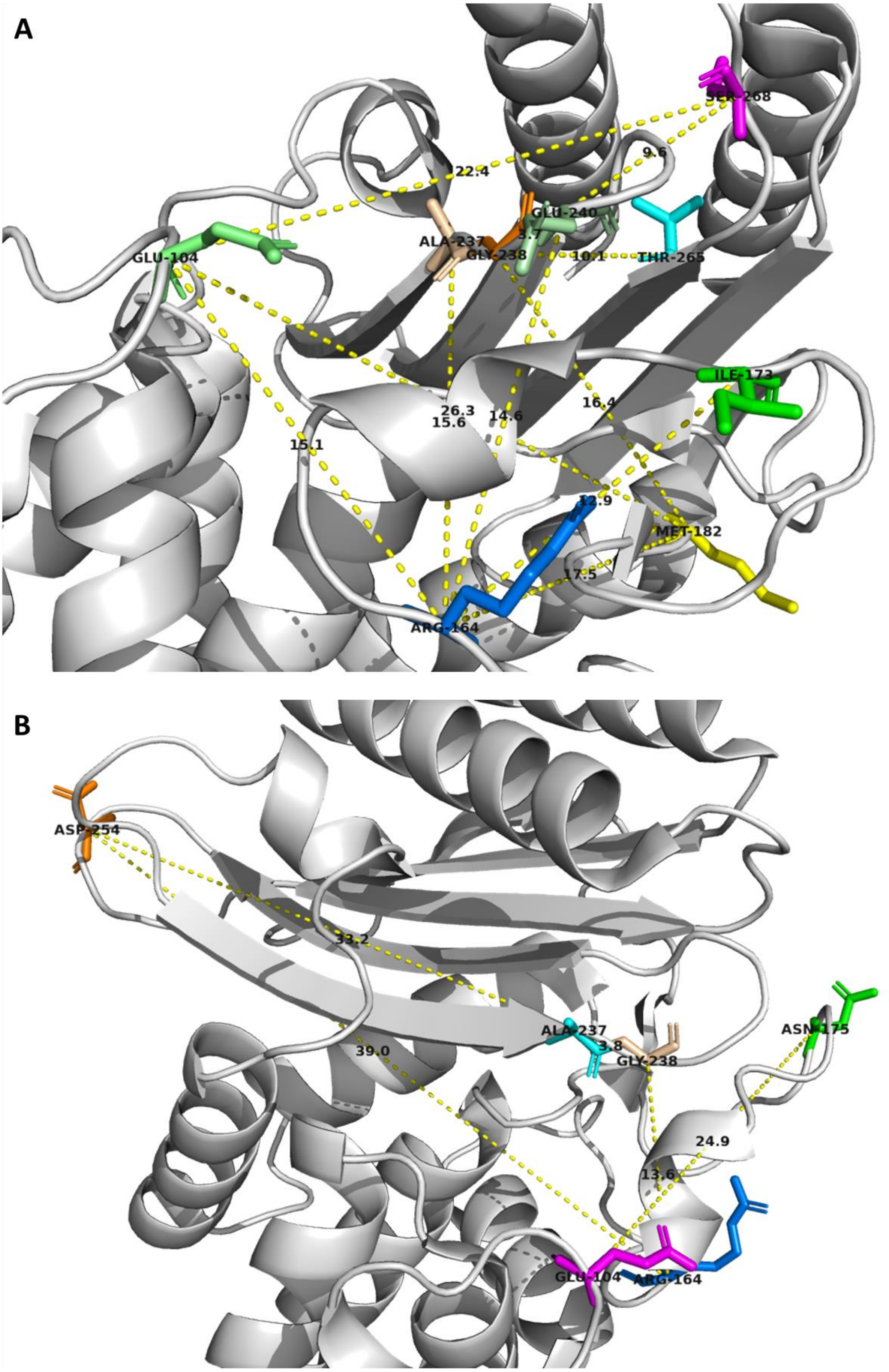


**Figure S3.** Distribution of residue positions resulting in a) positive and b) negative epistasis upon mutation. The illustration was generated using PyMOL 2.5.2 based on the PDB model 1zg4.

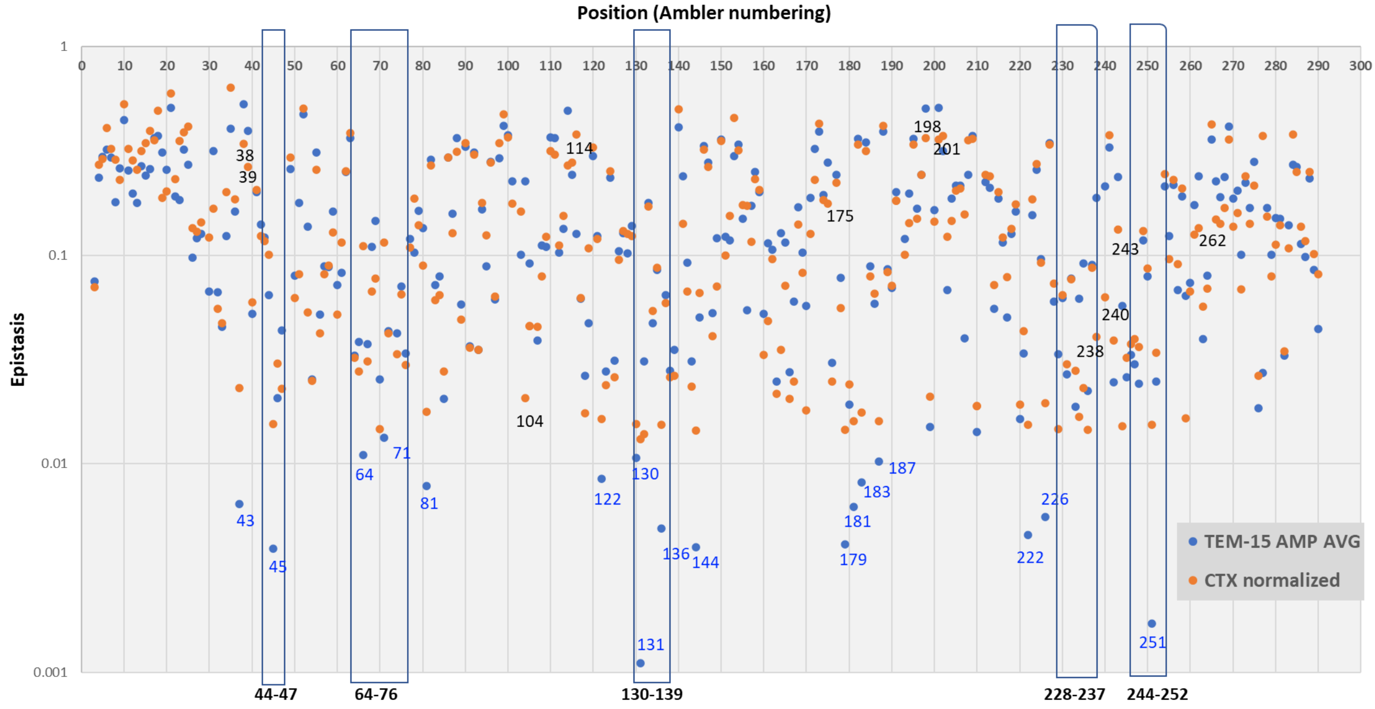


**Figure S4.** Average epistasis value for each position in TEM-15 (E104K_G238S) amino acid sequence as reported in [29]. Blue dots represent values obtained under ampicillin selection and red dots represent values obtained under cefotaxime selection. This figure supports the idea that the correlation between ampicillin and cefotaxime is driven by mutations that non-specifically decrease enzymatic activity, since both ampicillin and cefotaxime identify the critical areas for catalysis: positions 44 to 47, positions 64 to 76, positions 130 to 139, positions 228 to 237, and positions 244 to 252. Further, residues that show strongest epistasis under ampicillin selection are generally important for function. These include G45 and P183, which face each other and are strictly conserved; T71, which is essential for proper structural stability; S130 and D131, which are critical for catalysis (D131 also constrains H3 and H5 through strong anchoring interactions); D179, which forms a critical salt bridge with D164 that stabilizes the omega loop; M181 whose right orientation toward the aromatic ring of Y264 is essential; and R222, which forms a buried salt bridge with D233. On the other hand, residues that show strongest epistasis under cefotaxime selection (blue dots) include positions that are important for adaptation: 104, 238, and 240 as well as immediate neighbors: 241 and 198.

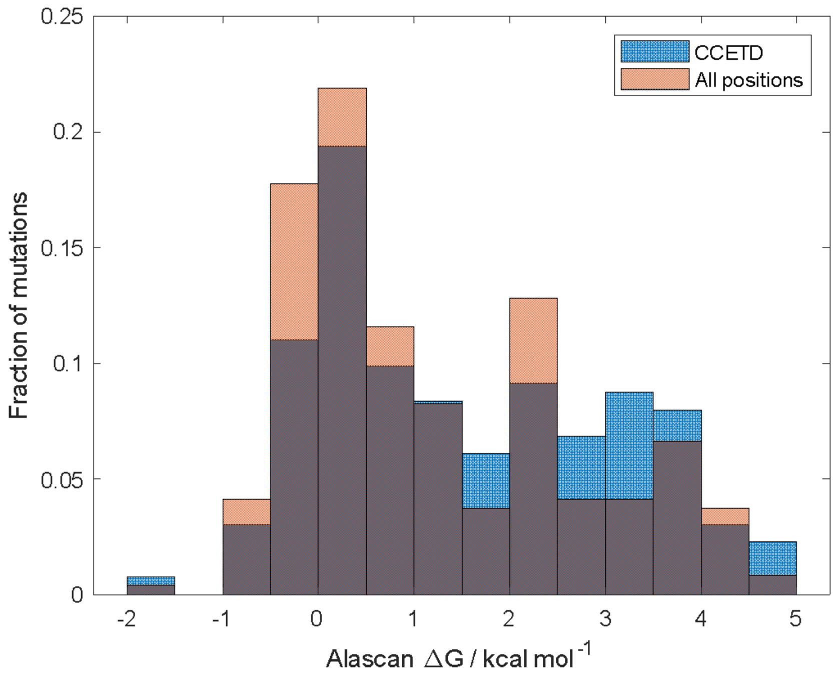


**Figure S5.** Histogram of the alanine scanning of all positions of TEM-1 (positions 26 to 289) vs. those positions that appear mutated in variants in the CCED database.

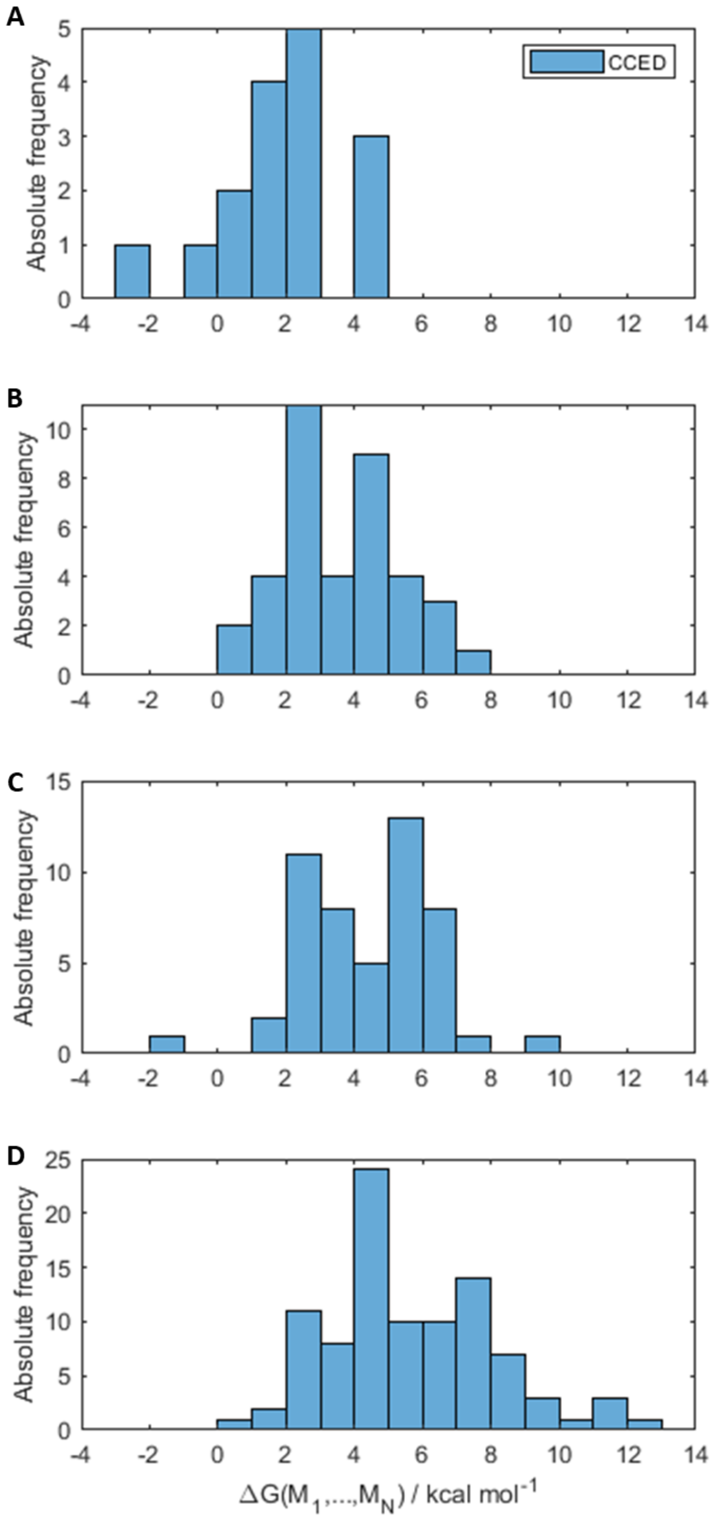


**Figure S6.** Relative frequency of different mutants in CCED containing a) 1, b) 2, c) 3, d) >3 mutations and their predicted change in the energy of folding relative to the wild type. The change in energy was computed by modeling all mutations simultaneously using FoldX. The results are in good agreement with those in Figure 3, which considers a linear approximation, confirming that the predicted effects of individual mutations on the free energy of folding in our system are roughly additive.

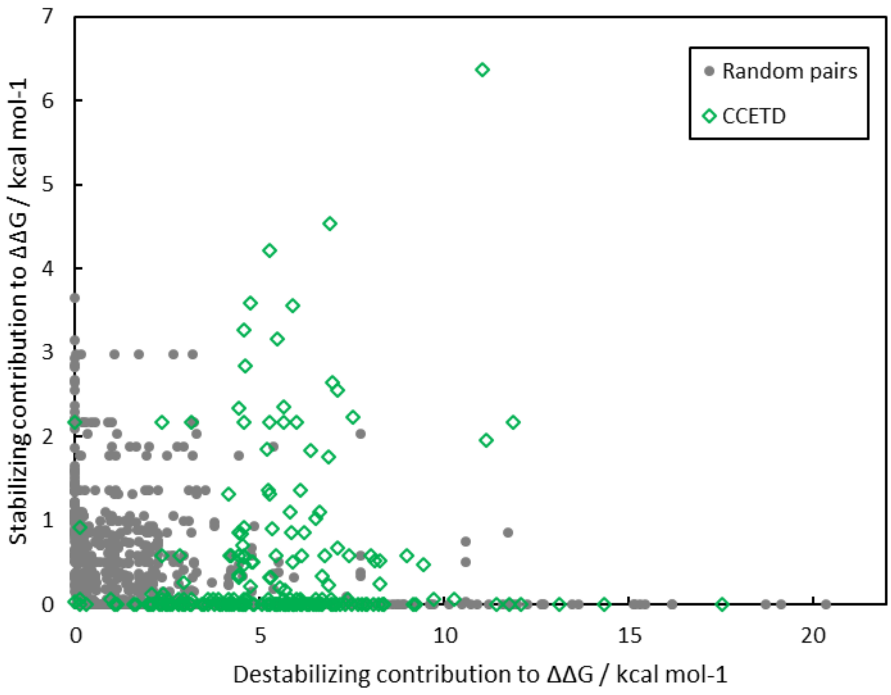


**Figure S7.** Comparison of stabilizing and destabilizing energy contributions in experimentally observed mutants (CCED), and in random pairs of individual mutations. The results suggest that strongly destabilizing mutations are more likely to be accompanied by stabilizing mutations.

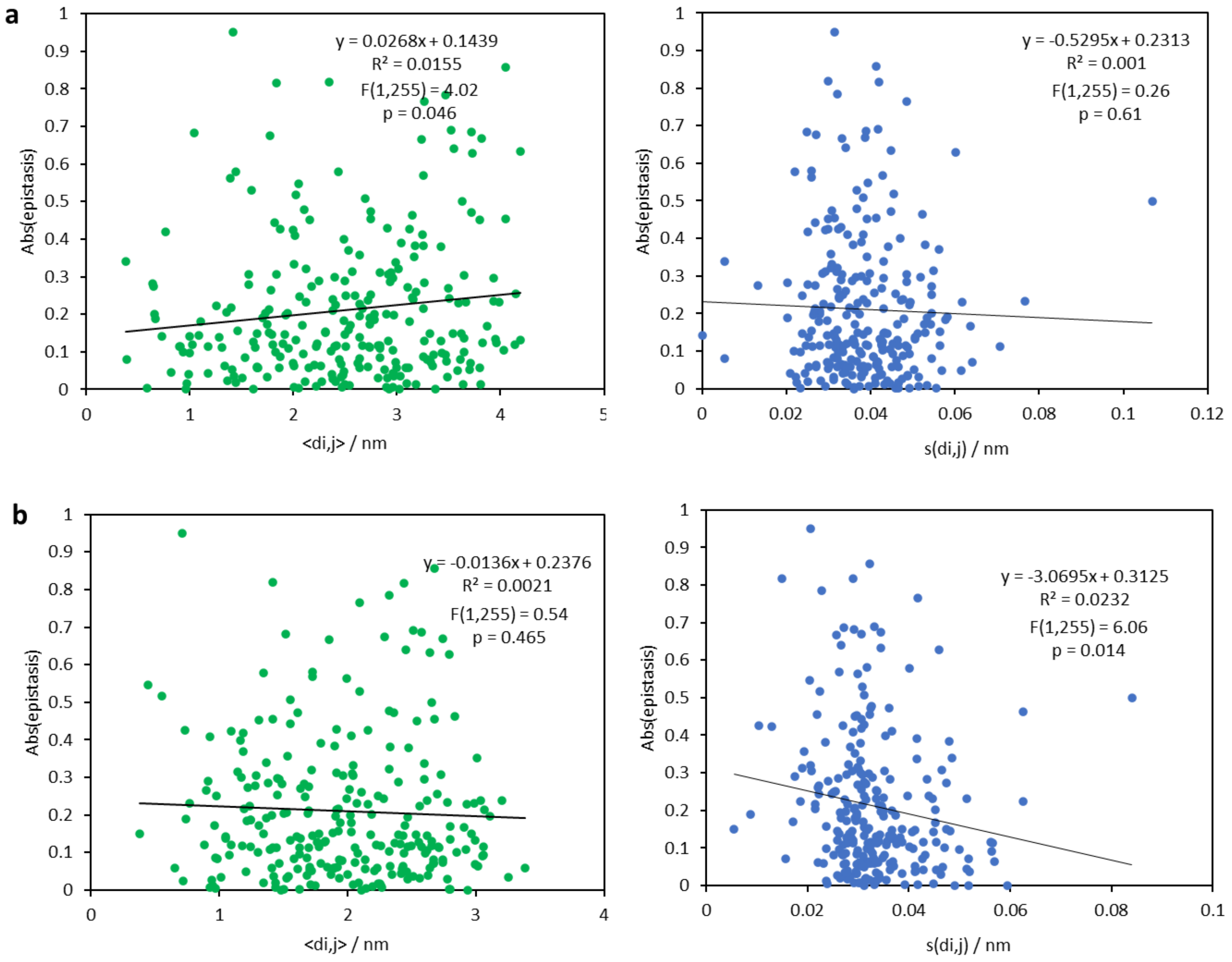


**Figure S8.** Relationship between the molecular dynamics descriptors proposed in this work and the magnitude of the epistasis in TEM-1 previously measured by Steinberg *et al*. using ampicillin [39]. For the amino acid positions considered (i and j), we evaluate their average distance, $\langle d_{i,j} \rangle$, as well the fluctuation of such distance over time, $s(d_{i,j})$, by carrying out MD simulations of the wild-type structure (PDB: 1ZG4). a) E104K and all other individual positions in TEM-1. b) G238S and all other individual positions in TEM-1. When multiple substitutions for a given pair of positions were evaluated, the average epistasis was computed for these plots. PDB model 1zg4 was used for computing the MD descriptors, as explained in the main text.
